# Genetic variation in *TMEM106B* alters microglial activation and cytokine responses in chronic traumatic encephalopathy

**DOI:** 10.1007/s00401-025-02955-7

**Published:** 2025-11-20

**Authors:** Shahar Hartman, Nurgul Aytan, Raymond Nicks, Samantha Hawkins, Jonathan Cherry, Victor E. Alvarez, Gaoyuan Meng, Yorghos Tripodis, Brett Martin, Joseph Palmisano, Lee E. Goldstein, Douglas I. Katz, Brigid Dwyer, Daniel H. Daneshvar, John F. Crary, Michael Alosco, Weiming Xia, Ann C. McKee, Jesse Mez, Thor D. Stein

**Affiliations:** 1 Boston University Alzheimer’s Disease Research Center and CTE Center, Department of Neurology, Boston University Chobanian & Avedisian School of Medicine, Boston, USA; 2 Department of Neurology, Boston University Chobanian & Avedisian School of Medicine, Boston, MA USA; 3 VA Bedford Healthcare System, Bedford, MA USA; 4 U.S. Department of Veteran Affairs, VA Boston Healthcare System, Jamaica Plain, 150 S. Huntington Avenue, Boston, MA 02130 USA; 5 Department of Pathology and Laboratory Medicine, Boston University Chobanian & Avedisian School of Medicine, Boston, MA USA; 6 Department of Biostatistics, Boston University School of Public Health, Boston, MA USA; 7 Biostatistics and Epidemiology Data Analytics Center, Boston University School of Public Health, Boston, MA USA; 8 Center for Health Data Science, Boston University School of Public Health, Boston, MA USA; 9 Department of Radiology, Boston University Chobanian & Avedisian School of Medicine, Boston, MA USA; 10 Department of Psychiatry, Boston University Chobanian & Avedisian School of Medicine, Boston, MA USA; 11 Departments of Biomedical, Electrical & Computer Engineering, Boston University College of Engineering, Boston, MA USA; 12 Braintree Rehabilitation Hospital, Braintree, MA USA; 13 Department of Physical Medicine and Rehabilitation, Harvard Medical School, Boston, MA USA; 14 Department of Pathology, Nash Family Department of Neuroscience, Department of Artificial Intelligence & Human Health, Ronald M. Loeb Center for Alzheimer’s Disease, Friedman Brain Institute, Icahn School of Medicine at Mount Sinai, New York, NY USA

**Keywords:** CTE, RHI, TMEM106B, Microglia, Cytokines, Neuropathology

## Abstract

Chronic traumatic encephalopathy (CTE) is a neurodegenerative disease caused by repetitive head impacts (RHI). However, individuals with similar RHI exposure can show differing pathology, suggesting a role for genetic variation. A common *Transmembrane Protein 106B* (*TMEM106B*) risk variant is associated with greater CTE severity, though its mechanism remains unclear. To determine whether *TMEM106B* alters the inflammatory response to pathology in CTE, we examined associations between microglia, via immunohistochemistry, and inflammatory cytokines, via immunoassay, in brain donors with CTE with and without the risk genotype (*rs3173615)*. We analyzed 323 RHI-exposed brain donors: 55 without pathology (controls) and 268 with CTE. Regression models tested associations between *TMEM106B* risk and CTE presence, CTE stage, TDP-43, and dementia in those < = 65 and > 65 years of age. Within a subset of 122 brain donors, we examined associations between microglia, cytokines, and pathology stratified by *TMEM106B* genotype. Among donors > 65 years old, the *TMEM106B* risk genotype was associated with increased CTE stage (OR = 2.748 [95% CI 1.183–6.383], *p* = 0.019), comparable to the effect of playing > 8 years of contact sports, and with greater odds of having TDP-43 inclusions (OR = 3.649 [95% CI 1.278–10.422], *p* = 0.016). In donors < = 65, *TMEM106B* risk was associated with higher odds of dementia (OR = 6.912 [95% CI 2.015–23.705], *p* = 0.002). *TMEM106B* gene variation had a significant effect on associations between inflammatory markers and CTE-related pathology. In the protective genotype, IL-8 and IL-6 demonstrated positive associations with CD68, TREM2, and tau pathology within the dorsolateral prefrontal cortex. In the risk genotype, IFN-γ, IL-4, TNF-α, TNF-β, and IL-10 demonstrated negative associations with TREM2 (*p*’s < 0.05), and TNF-α was negatively associated with cortical tau (*p* = 0.003). These results suggest that the microglial production of TREM2-associated cytokines and their association with pathology is aberrant in the *TMEM106B* risk genotype in CTE. Overall, *TMEM106B rs3173615* is associated with an increased risk of developing higher stage CTE and TDP-43 pathology, potentially via impaired microglial activation and aberrant cytokine production.

**Supplementary Information:**

The online version contains supplementary material available at 10.1007/s00401-025-02955-7.

## Introduction

Chronic traumatic encephalopathy (CTE) is a neurodegenerative disease caused by repetitive head impacts (RHI), which frequently occur in contact/collision sports, military service, and physical violence [7, 38, 48]. Currently, CTE refers to the post-mortem neuropathology, and Traumatic Encephalopathy Syndrome (TES) encompasses the clinical syndrome. TES includes cognitive impairment and/or neurobehavioral dysregulation as core clinical features [24]. Pathologically, CTE is a progressive tauopathy defined by intraneuronal hyperphosphorylated tau (p-tau) inclusions within neurons and sometimes glial cells around small blood vessels and concentrated at the cortical sulcal depths [6, 33]. CTE is frequently associated with other pathological inclusions, including TAR DNA-Binding Protein 43 (TDP-43) [36, 47]. CTE has been stratified into four distinct pathological stages characterized by greater regional involvement and increasing p-tau severity. Stage I typically involves one or two isolated foci of p-tau neurofibrillary tangles (NFT) with clustered dot-like neurites surrounding small blood vessels at the sulcal depths in the frontal cortex. Stage II is characterized by clusters of NFTs in multiple cortical lobes. In stage III, p-tau inclusions are present in multiple cortical lobes as well as the medial temporal lobe, brainstem, striatum, thalamus, and mamillary body. Stage IV, the most severe, demonstrates abundant NFTs in the cortex with prominent degeneration of the medial temporal lobe and involvement of the diencephalon, brain stem, cervical spinal cord, and the dentate nucleus of the cerebellum [1, 35, 37, 39]. The total years of contact sports play and age are significant predictors of both developing CTE and with increasing disease stage, and recent data suggest that accelerometer estimates of forces broken down by position player further improve the prediction of CTE pathology in American football [16]. However, significant variation in CTE severity still exists even accounting for RHI exposure and age. Genetic variation may underlie this heterogeneity and common genetic variation in both the *apolipoprotein ε (APOE)* gene and the *transmembrane protein 106B gene (TMEM106B)* have been associated with greater CTE stage [4, 12].

Genetic variation in *TMEM106B* is a risk factor for multiple diseases, including frontotemporal lobar degeneration with TPD-43-immunoreactive pathology (FTLD-TDP), limbic-predominant age-related TDP-43 encephalopathy (LATE), and AD in *APOE ε4* negative individuals [18, 22, 58]. We recently reported an association between *TMEM106B* risk and greater CTE severity [11]. However, the mechanism behind this association is unknown. *TMEM106B* is a gene that codes for a type II glycosylated transmembrane protein that localizes to late lysosomes. The gene has been implicated in controlling the size, shape, and acidification of the lysosome in microglia [8]. The single-nucleotide polymorphism (SNP) *rs3173615* occurs within the coding region of *TMEM106B* and may alter the stability of the protein product [45] leading to a dysfunctional lysosome, increased oxidative stress and cytotoxicity [12]. Microglia play a prominent role in the clearance of multiple pathologies and their dysfunction is linked to multiple neurodegenerations [19]. Microglial activation may promote tau accumulation in CTE [13].

Here, we set out to test associations between the T185 (risk) variant at *rs3173615* with CTE status, stage, TDP-43 inclusions, and dementia. We further examine associations with microglia density and phenotypes, pro- and anti-inflammatory cytokines, and pathology in the absence and presence of *TMEM106B* risk. Overall, we find that *TMEM106B* risk is associated with an increased risk of developing higher stage CTE and TDP-43 pathology and has distinct effects on cytokines associated with activated microglia and tau pathology.

## Materials and methods

### Participants

**Fig. 1:**
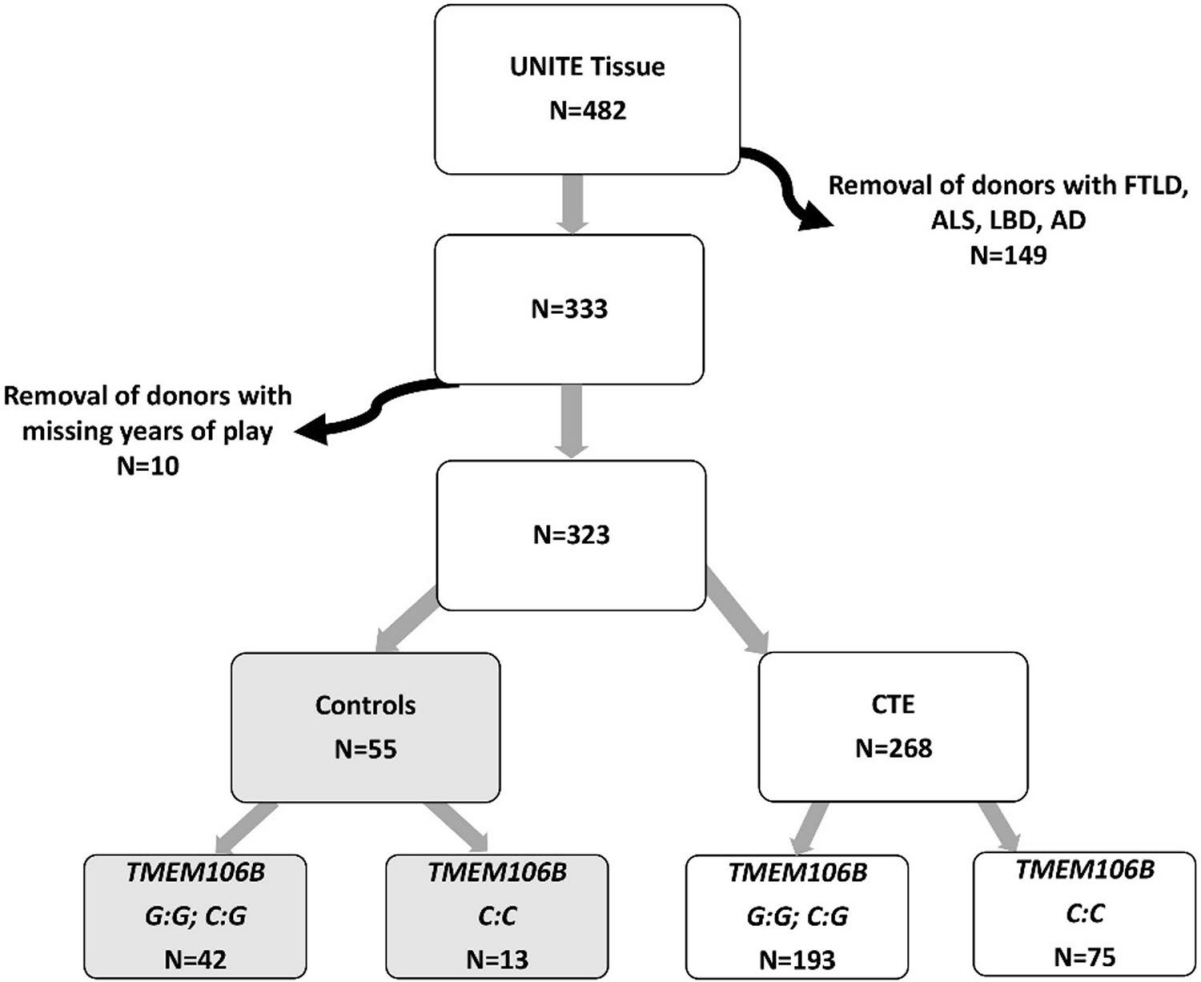
Flowchart showing participant inclusion. Curved arrows indicate excluded participants. Of the 482 donors, 149 donors were removed because of FTLD, ALS, LBD, and AD pathology. 323 donors without significant comorbid pathology remained, including 55 without CTE (controls) and 268 with CTE. *CTE* chronic traumatic encephalopathy, *UNITE* understanding neurologic injury and traumatic encephalopathy

Four hundred and eighty-two brain donors with a history of RHI and *TMEM106B* genotypes were available for study from the Understanding Neurologic Injury and Traumatic Encephalopathy (UNITE) Brain Bank [41]. Donors with frontotemporal lobar degeneration (FTLD), amyotrophic lateral sclerosis (ALS), neocortical Lewy body disease (LBD), and Alzheimer’s disease (AD) (*n* = 149) were excluded as *TMEM106B* has been separately implicated in these other neurodegenerative conditions [12]. Ten donors had missing data on years of play and were excluded. Of the remaining 323, there were 268 donors with CTE and 55 without any significant neurodegenerative pathology (controls; Fig. 1). We conducted a supplementary analysis in a cohort of donors with Alzheimer’s disease only (*n* = 51; G:G/G:C = 35; C:C = 16; median age [IQR] = 89.0 [10]). A table depicting the Demographic, Clinical, Genetic, and Neuropathological Characteristics for AD cohort is provided in Supplementary table a. Consent for brain donation and research participation were provided by donor next-of-kin. An authorized legal representative provided written consent for participation and brain donation. IRB approval for the brain donation program was obtained through the Boston University Alzheimer’s Disease & CTE Center and the VA Bedford Healthcare System.

### Neuropathological assessment

Neuropathological assessment followed the Understanding Neurologic Injury and Traumatic Encephalopathy (UNITE) brain bank procedures as described previously [29, 39, 56]. Briefly, sections were stained for Luxol fast blue, hematoxylin and eosin (LHE), Bielschowsky’s silver, phosphorylated tau (ptau) (AT8), phosphorylated TDP-43, alpha-synuclein, and amyloid-β (Aβ) to perform complete neuropathological diagnoses [34]. TDP-43 inclusions were assessed within the amygdala, hippocampus, and dorsolateral frontal cortex and were marked as positive for TDP-43 pathology if present in any of those regions. The neuropathological diagnosis of CTE was made blinded to the donor’s clinical history using the NINDS consensus criteria for CTE that require abnormal perivascular accumulations of hyperphosphorylated tau in neurons in an irregular and patchy distribution [6, 33]. CTE was staged based on regional involvement and severity of p-tau inclusions using validated criteria [1, 6].

### Genotyping

DNA was extracted from participant fresh-frozen brain tissue using the Promega Maxwell RSC Tissue DNA Kit (Cat No# AS1610) according to the manufacturer’s protocol. Genotyping of *TMEM106B rs3173615* variant was performed using TaqMan SNP genotyping assay (Applied Biosystems) on the StepOnePlus Real-Time PCR system to determine three different genotype (GG, CG, and CC). The risk genotype was defined as the homozygous “C” genotype, and the protective genotype is defined as either the “GG” or the “CG” genotypes. The genotyping of *APOE* was performed using TaqMan assays (Applied Biosystems) at two single-nucleotide polyvariations, *rs429358 and rs7412*, to determine six possible *APOE* genotypes (ε2/ε2, ε2/ε3, ε2/ε4, ε3/ε3, ε3/ε4, and ε4/ε4).

### Immunoassay and immunohistochemical study subset

A subset of brain donors diagnosed with CTE or AD and possessing both frozen and formalin-fixed tissue from the dorsolateral prefrontal cortex (DLPFC, Brodmann area 8/9) collected by December 1, 2021 were included for analysis. This subset enabled parallel investigation using quantitative immunoassay methods on frozen tissue and immunohistochemical analyses on fixed tissue from the same donors.

### Quantitative immunoassay (frozen tissue)

Frozen DLPFC tissue from the selected donors was used for quantitative immunoassay analysis, as such methods require fresh-frozen material. Analyses included measurement of neurodegeneration-related markers (total tau, phosphorylated tau at residues 181, 202, 231, and 396, and beta-amyloid 40 and 42), and inflammatory cytokines, following previously described protocols [46, 48].

Briefly, frozen tissue was homogenized in freshly prepared, ice-cold 5 M guanidine hydrochloride in Tris-buffered saline (20 mM Tris–HCl, 150 mM NaCl, pH 7.4) with protease and phosphatase inhibitors at a 5:1 ratio of buffer to tissue (ml:g) using a Qiagen Tissue Lyser LT at 50 Hz for 5 min. Homogenates were mixed (regular rocker) overnight at room temperature. The lysate was diluted with 1% Blocker A (Meso Scale Discovery (MSD), Rockville, Maryland, #R93BA-4) in wash buffer according to specific immunoassays: 1:300 for total tau, pTau231 (MSD #K15121D-2), and pTau181, 1:100 for PSD-95 (MSD #K150QND), and 1:80 for beta-amyloid (MSD #K15200E-2). Samples were subsequently centrifuged at 17,000g and 4°C for 15 min, after which the supernatant was applied to the immunoassays. To capture tau phosphorylated at Thr residue 181, antibody AT270 was used and the detecting antibody was the biotinylated HT7 that recognizes residue 159–163 of tau (Thermo Scientific, Rockford, IL). Internal calibrators of p-tau and tau were used (MSD) [11]. Standards with known concentrations were used for Aβ. For PSD-95, arbitrary values were assigned to a reference brain lysate, which was run as a standard curve with every plate. All standards and samples were run in duplicate.

For neuroinflammatory cytokine measurements, tissue was diluted in ice-cold RIPA buffer (Thermo Scientific) at a 5:1 (RIPA volume in mL: brain wet weight in grams) and homogenized with Qiagen Tissue Lyser LT, at 50 HZ for 5 min. Samples were then centrifuged at 17,000 g, 4 °C, for 15 min. Two different panels were used: Proinflammatory Panel 1 and Cytokine Panel 1. Proinflammatory Panel 1 consisted of microplates pre-coated with capture antibodies for IFN-γ, IL-1β, IL-2, IL-4, IL-6, IL-8, IL-10, IL-13, and TNF-α. Cytokine Panel 1 consisted of microplates pre-coated with capture antibodies for IL-1α, IL-5, IL-7, IL-12/IL-23p40, IL-15, IL-16, IL-17A, TNF-β, and VEGF. However, we focused on IL-1α and TNF-β due to their a priori involvement in neurodegenerative disease. The antibodies were conjugated with an MSD SULFO-TAG and the MSD platform detected the sulfo-tag to provide a quantitative measure of the analytes in the sample. TREM2 was measured with an enzyme-linked immunoassay kit (Life Span Bio, LS-F35073). RIPA lysate was diluted 1:10 by dilutant A of the kit and applied to the assay according to the manufacturer’s instruction.

### Immunohistochemistry (fixed tissue)

Formalin-fixed, paraffin-embedded tissue blocks from the same subset of donors examined by immunoassay were used for immunohistochemical analysis. Sections from the DLPFC were cut at 10 μm for hyperphosphorylated tau (AT8; Invitrogen MN1020; 1:1000) staining, and at 20 μm for IBA1 (Wako 019–19741; 1:500) and CD68 [KP1] (Vector Laboratories VP-C364; 1:1000) staining, each following established protocols (Mez et al., 2015). Following staining, all sections were scanned at 20 × magnification using a Leica Aperio Scanscope (Leica Biosystems, Richmond, IL). Digital images were subsequently reviewed and analyzed using Aperio eSlide Manager and ImageScope software (Leica Biosystems).

By restricting analyses to cases where both frozen and fixed DLPFC tissues were available, we ensured that quantitative immunoassays and immunohistochemical studies were conducted on matched anatomical regions from the same donors. This approach strengthened comparative analysis of molecular and histopathological findings within individual brains.

### Digital microscopy and analysis

Whole stained DLFC sections were scanned and digitized using an Aperio ScanScope AT Turbo. Digital images of IBA1 and CD68 were viewed and analyzed using Aperio ImageScope (Leica). Analysis of digital images were limited to the depth of the superior frontal sulcus which was denoted as the bottom third of the connecting superior and middle gyri. The white matter–gray matter boundary was used as the edge of the region of interest to ensure that only gray matter was highlighted. This allowed for the white matter to be excluded. The Aperio nuclear algorithm (Version 9) was set to recognize and count IBA1 and CD68 positive cells.

**Fig. 2:**
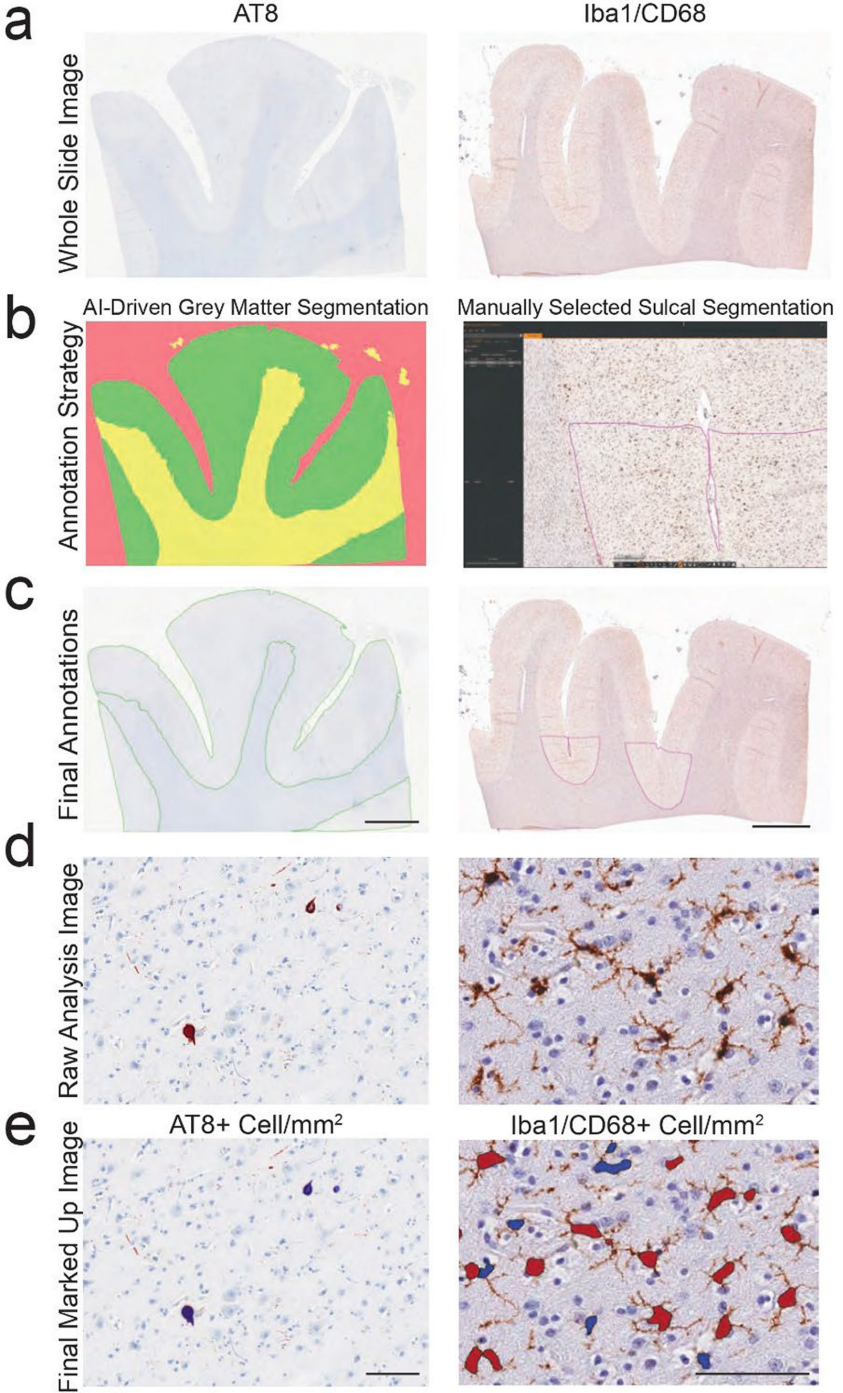
Example of digital pathology analysis workflow. **a** Glass slides stained with AT8, Iba1, or CD68 were digitized with Aperio Slide Scanners to create whole slide images (WSI). **b**, **c** WSI were annotated with two strategies. For AT8, an HALO AI algorithm was created to automatically identify gray (green) vs white (yellow) matter, and glass (red). For Iba1/CD68, sulcal annotations were manually drawn. Scale bar = 5 mm. **d**, **e** Representative example of the output for quantitation algorithms for AT8 (Left) and Iba1/CD68 (Right), and resulting markup images denoting positive cells from background/non-positive cells. **e** Blue markup denotes AT8 + cell in left panel and brown markup denotes positive cells in right panel. Scale bar = 100 um

For the analysis of AT8 + cell densities, three AI algorithms were developed and trained using Indica Laboratories HALO. First, we trained a Densenet AI algorithm to recognize gray matter, white matter, and glass. 110 annotations across 19 images were used for model training with 212,910 iterations, achieving a cross-entropy of 0.137 with a resolution of 1.44 µm pixel—1, and a minimum object size set to 0.5 mm^2^. The finished classifier was then used to automatically annotate gray matter. Annotations were thoroughly reviewed by a trained human observer alongside a board-certified neuropathologist to ensure accuracy in identifying the intended structures or cell types. Once the gray matter was correctly annotated, a second AI algorithm was trained to recognize gray matter AT8 + cells. The Nuclear Segmenter classifier was trained to recognize AT8-positive cells, and then, the Object Phenotyper classifier was trained on 2555 AT8-positive cells. The model was allowed to iterate 1,300,760 times and achieved a cross-entropy less than 0.005. The final count of AT8 + cells was then standardized to the gray matter area. Values are presented at AT8 + cell/mm2. This quantification method has been previously described and validated (see Fig. 1 of [23]). An example of the digital pathology analysis is provided in Fig. 2.

### Retrospective clinical assessments

Retrospective clinical data were collected through online surveys, telephone interviews with next-of-kin, and by reviewing clinical records. Neuropathologically findings were not shared with the interviewer or the interviewees. The interviews were conducted by clinicians with expertise in adapting standardized clinical scales for retrospective post-mortem administration. Next of kin provided a contact sport history, including levels played, years played at each level, and age of first exposure. Antemortem dementia status (present/absent) was determined through a diagnostic consensus conference of expert clinicians who were blinded to brain donors’ pathological diagnoses. A consensus diagnosis of dementia was adjudicated based on modified Diagnostic and Statistical Manual of Mental Disorders (Fourth Edition, Text Revision) criteria [3]. The diagnosis was informed by the structured interview conducted by research assistants, the unstructured interview conducted by clinicians, as well as the online surveys [41].

### Statistical analysis

Separate multivariate binary logistic regressions were performed in age-stratified groups to test whether *TMEM106B* risk genotype (CC) was associated with the neuropathological diagnosis of CTE, the presence of TDP-43 inclusions, or dementia. A multivariable ordinal logistic regression model was performed in age-stratified groups to test whether *TMEM106B* risk genotype (CC) was associated with CTE stage. We selected age 65 for age-stratified analyses, because this cutoff was used previously to determine age-dependent effects of APOE genotype, is frequently used to distinguish early- from late-onset dementia, and is close to the median age in this sample thus providing similar power to detect an association in each group [4]. All models were adjusted for age of death and the number of years of contact sports played. Table 2 was adjusted for age of death, the number of years of contact sports played, *APOE4* status, and self-reported African-American race. To express the *TMEM106B* effect in equivalent “years of contact sports,” we computed the logarithmic ratio of effect sizes, using the effect of years played as the base and the *TMEM106B* effect as the argument. This was performed separately for CTE stage and for dementia status. Effect sizes for the ordinal logistic regression model predicting CTE stage (CTEStage) in participants aged > 65 years were estimated using SPSS and G*Power (version 3.1.9.7). Pseudo-*R*^2^ values were calculated based on the -2 Log Likelihood of the full and intercept-only models ([15, 42]. Cohen’s *f*^2^ was derived from the Nagelkerke *R*^2^ (*f*^2^ = *R*^2^/[1−R^2^]) to estimate the proportion of variance explained by the predictors for power analysis [14]. For the present model (*N* = 110, 5 predictors), Nagelkerke *R*^2^ = 0.212, yielding *f*^2^ ≈ 0.268 and indicating high statistical power (1–β ≈ 0.993, computed with GPower). Full details of these calculations are provided in the Supplementary figure b. Cytokine measures exhibited skewness and were normalized using a rank-based normalization method [53]. Multiple linear regressions testing the associations of both anti-inflammatory (IL4, IL8, IL10, IL13) and pro-inflammatory (IFN-γ, IL1A, IL1B, TNF-α, TNF-β, IL6) cytokines with markers of beta-amyloid, phosphorylated tau were performed separately in those with and without the *TMEM106B* risk genotype (CC) and adjusted for age at death and APOE ε4. The effects seen remained the same when adjusting for duration of play in a subsequent analysis. A sensitivity analysis for the multiple linear regressions was conducted in a set of Caucasian donors in the protective genotype as our previous study included only Caucasian donors. This analysis was only conducted in the protective genotype as there were no African-American donors in the risk genotype cohort. To distinguish the effects seen in CTE from AD, we further conducted the same main analysis in an AD-only cohort and adjusted for age at death, APOE ε4, and sex. The corrections for multiple comparisons were conducted using a False Discovery Rate (FDR) with alpha set to 0.05 [5]. Statistical analyses were performed using SPSS (v.27, IBM). Heatmaps were created using Prism (version 9.1.2, Graphpad Software).

## Results

Of the 323 male brain donors with a history of RHI from contact or collision sports or military service, the median (IQR) age was 60 (42.0–72.0) and 58 (18.2%) were Black. The sample included 268 brain donors with neuropathologically confirmed CTE pathology (83.0%) and 55 brain donors without evidence of any significant neurodegenerative pathology (17.0%). Of 268 donors with CTE, 73 (27.2%) had stage I CTE, 56 (20.9%) had stage II, 100 (37.3%) had stage III, and 39 (14.6%) had stage IV. TDP-43 inclusions were often present in those with stage III and IV CTE. Dementia was also more common among those with high stage CTE (Table 1).

**Table 1:** Demographic, clinical, genetic, and neuropathological characteristics stratified by CTE control status and CTE stage

Characteristic	Controls (*n* = 55)	All CTE (*n* = 268)	Total (*n* = 323)	< = 65 years old (*n* = 138)	> 65 years old (*n* = 110)
Sex (male/female/other)	54/0/1	268/0/0	322/0/1	137/0/1	110/0/0
Age median (IQR),	54.0 (27.0–68.0)	62.0 (44.0–73.0)	60.0 (42–72)	46.0 (27–57.0	74.0 (70.0–80.0)
Self-reported Black race (%)	9.1% (5)	20.1% (53)	18.2% (58)	23.9% (33)	10.1% (11)
Self-reported White race (%)	87.3% (48)	77.7% (205)	79.3% (253)	72.5% (100)	89.9% (98)
RHI years median (IQR)	11.0 (6.0–13.0)	15.0 (12.0–19.0)	14.0 (10.0–19.0)	14.0 (10.0–18.0)	14.0 (11.0–20.0)
Age of first contact sports exposure median (IQR)	10.0 (7.0–13.0)	10.0 (8.0–13.0)	10.0 (8.0–13.0)	9.0 (6.0–12.0)	13.0 (10.0–14.0)
Military exposure %Y (Y/N)	22.6% (12/41)	18.7% (49/213)	19.4% (61/254)	9.6% (13/122)	35.2% (37/68)
**Sport**					
Football	44 (80.0%)	243 (90.7%)	287 (88.8%)	119 (86.2%)	103 (93.6%)
Hockey	2 (3.6%)	9 (3.3%)	11 (3.4%)	7 (5.1%)	2 (1.9%)
Other	9 (16.4%)	16 (6.0%)	25 (7.7%)	26 (19.0%)	5 (4.5%)
*TMEM106B* (CC**/**G +)-	13/42 (23.6%)	75/193 (28.0)	88/235 (27.2%)	36/102 (26.1%)	30/80 (27.3%)
*APOE E4 *status* % (Y/N)*	23.6% (13/42)	30.6% (59/134)	29.0% (72/176)	29.0% (40/98)	29.1% (32/78)
TDP-43% (Y/N)	3.9% (2/49)	23.2% (59/195)	20.0% (61/244)	4.3% (6/120)	46.7% (49/56)
Dementia status % (Y/N)	13.0% (7/47)	46.7% (121/138)	40.9% (128/185)	21.7% (30/105)	69.9% (72/31)
**CTEStage**					
No CTE	56 (100%)	0 (0%)	55 (17.0%)	38 (27.5%)	17 (15.5%)
Low	0 (0%)	129 (48.1%)	129 (39.9%)	65 (37.1%)	19 (17.3%)
High	0 (0%)	139 (51.9%	139 (43.1%)	35 (25.4%)	74 (67.2%)
Brainstem Lewy body pathology %Y (Y/N)	0% (0/55)	2.2% (6/262)	1.9% (6/98.1)	0.7% (1/137)	1.8% (2/108)
**Alzheimer disease neuropathologic change**					
* Amyloid score*					
A0	33 (68.8%)	134 (58.8%)	167 (60.5%)	96 (79.3%)	30 (31.3%)
A1	9 (18.8%)	43 (18.9%)	52 (18.8%)	18 (14.9%)	26 (27.1%)
A2	4 (8.2%)	23 (10.1%)	27 (9.8%)	6 (5.0%)	16 (16.7%)
A3	2 (4.2%)	28 (12.2%)	30 (10.9%)	1 (.8%)	24 (24.9%)
*Braak stage*					
0	33 (68.8%)	58 (25.6%)	91 (33.0%)	67 (55.4%)	5 (5.2%)
I–II	8 (16.7%)	72 (31.7%)	80 (29.0%)	32 (26.4%)	19 (19.8%)
III–IV	6 (12.4%)	90 (39.6%)	96 (35.1%)	21 (17.4%)	66 (68.8%)
V–VI	1 (2.1%)	7 (3.1%)	8 (2.9%)	1 (.8%)	6 (6.3%)
* CERAD score*					
C0	43 (89.6%)	191 (83.8%)	234 (84.8%)	119 (98.3%)	62 (64.6%)
C1	5 (10.4%)	37 (16.2%)	42 (15.2%)	2 (1.7%)	34 (35.4%)
C2	0 (0%)	0 (0%)	0 (0%)	0 (0%)	0 (0%)
C3	0 (0%)	0 (0%)	0 (0%)	0 (0%)	0 (0%)
Primary age-related tauopathy %Y (Y/N)	20.8% (10/38)	56.1% (128/100)	50.0% (138/276)	42.1% (51/121)	57.3% (55/96)
Atherosclerosis (moderate–severe) %Y (Y/N)	38.3% (18/29)	34.4% (76/145)	35/1% (94/174)	17.1% (20/97)	66.3% (63/32)
Arteriolosclerosis (moderate–severe) %Y (Y/N)	60.4% (29/19)	72.2% (164/63)	70.2% (193/82)	55.4% (67/54)	93.7% (89/6)
Remote microinfarcts % (Y/N)	10.4% (5/43)	19.7% (45.183)	18.1% (50/226)	5.0% (6/115)	35.4% (34/62)
Cerebral amyloid angiopathy (moderate-severe)	4 (8.4%)	29 (12.8%)	33 (11.9%)	3 (2.5%)	24 (25.0%)
*White matter rarefaction*					
None	18 (38.3%)	44 (19.6%)	62 (22.8%)	44 (36.7%)	4 (4.3%)
Mild	15 (31.9%)	95 (42.4%)	110 (40.4%)	49 (40.8%)	36 (38.3%)
Moderate	12 (25.5%)	65 (28.9%)	77 (28.3%)	20 (16.7%)	43 (45.7%)
Severe	2 (4.3%)	21 (9.3%)	23 (8.5%)	7 (5.8%)	11(11.7%)

Within the full sample, there were no significant *TMEM106B* associations for CTE status, CTE stage, or dementia. A previous study demonstrated age-dependent effects of the APOE genotype with a stratified age analysis at age 65; therefore, we ran analyses stratified by age-group (< = 65 years, n = [138] vs > 65 years, *n* = [110]) [4]. A binary logistic regression adjusted for age at death, years of playing contact sports, *APOE4* status, and African-American race did not show a significant association between the *TMEM106B* risk genotype and CTE status in those below or above 65 years of age. However, ordinal logistic regression in donors > 65 years old demonstrated that the *TMEM106B* risk genotype was associated with increased CTE stage (OR = 2.748 [95% CI 1.183–6.383], *p* = 0.019). The effect size for the *TMEM106B* risk genotype with the progression of CTE stage was similar to playing more than 8 years of contact sports. *TMEM106B* risk genotype was also associated with greater odds of having TDP-43 inclusions in those > 65 years (OR = 3.649 [95% CI 1.278–10.422], *p* = 0.016). In those < = 65 years of age, *TMEM106B* risk was associated with greater odds of having dementia (OR = 6.912 [95% CI 2.015–23.705], *p* = 0.002; Table 2).

**Table 2:** Associations of *TMEM106B* risk allele status with CTE status, CTE stage, TDP-43, and dementia status

	Age < = 65 (*n* = 138)			Age > 65 (*n* = 110)		
	OR	95% CI	*P*	OR	95% CI	*P*
CTE status						
* TMEM106B*	1.866	0.696–5.001	0.215	1.643	0.424–6.369	0.472
Duration of play, yrs	**1.139**	**1.050–1.234**	**0.002**	**1.196**	**1.062–1.348**	**0.003**
Age	1.010	0.984–1.038	0.451	1.059	0.969–1.157	0.208
CTE stage						
* TMEM106B*	1.105	0.528–2.313	0.792	**2.748**	**1.183–6.383**	**0.019**
Duration of play, yrs	**1.091**	**1.041–1.144**	** < 0.001**	**1.120**	**1.053–1.192**	** < 0.001**
Age	**1.047**	**1.023–1.070**	** < 0.001**	**1.060**	**1.007–1.115**	**0.027**
TDP-43 inclusions						
*TMEM106B*				**3.649**	**1.278–10.422**	**0.016**
Duration of play, yrs				**1.111**	**1.029–1.199**	**0.007**
Age				**1.073**	**1.005–1.145**	**0.034**
Dementia						
*TMEM106B*	**6.912**	**2.015–23.705**	**0.002**	1.405	0.467–4.225	0.545
Duration of play, yrs	**1.073**	**1.004–1.147**	**0.037**	1.009	0.945–1.078	0.787
Age	**1.100**	**1.049–1.154**	** < 0.001**	**1.194**	**1.088–1.309**	** < 0.001**

Binary logistic regression results for CTE status, TDP-43, dementia status, and ordinal logistic regression for CTE stage (0–IV). All models were adjusted for age at death, years of playing contact sports, *APOE4* status, and self-reported African-American race. Significant results are bolded. Sample size for the presence of dementia and the presence of TDP-43 row differed from CTE status and CTE stage [Age < = 65_dementia_ (135), Age > 65_dementia_ (103), Age < = 65_TDP-43_ (126), Age > 65_TDP-43_ (105)]. Effect size for the ordinal logistic regression model was computed and demonstrated a post hoc power ≈ 0.993 (G*Power 3.1.9.7)

**Fig. 3:**
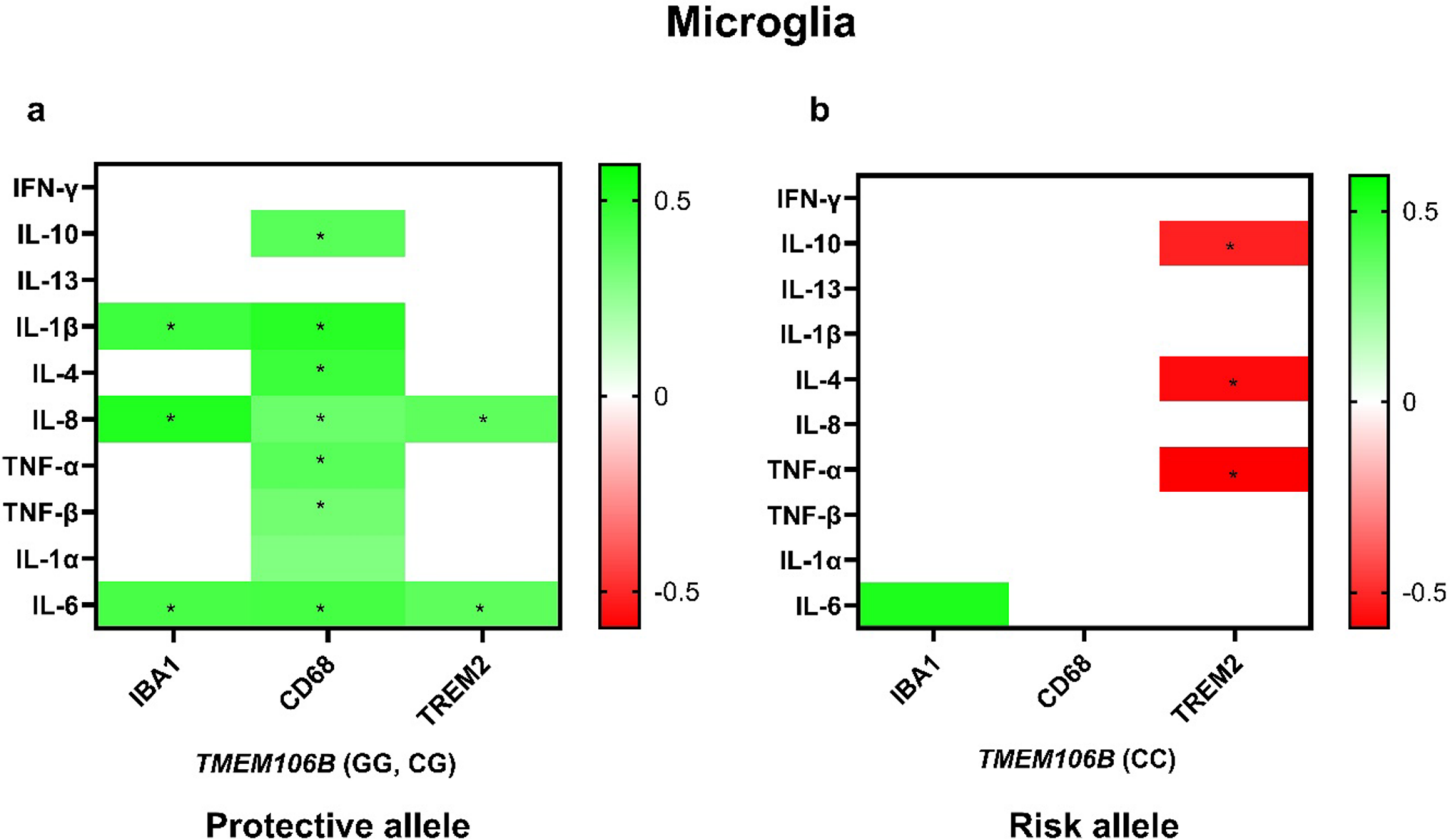
Effect of *TMEM106B* gene variation on microglial levels in CTE. Cytokine associations with microglia subtypes vary by *TMEM106B* risk in CTE. Multiple linear regression analyses adjusted for age and APOE ε4 tested associations between inflammatory cytokines and microglial markers within the protective genotype (**a**) and the risk genotype (**b**). Heatmaps show *β* values with *p* < 0.05 (red-to-green shading indicated by key). *p < 0.05 after FDR adjustment

A subset of brain donors with CTE who had both fixed and frozen tissue from the dorsolateral prefrontal gray matter were included in the analysis. These samples were evaluated using a panel of neurodegeneration-related markers, and inflammatory cytokines. Fixed tissue was used for immunostaining, while immunoassay measurements required frozen tissue. Only the ratio of Ptau_202_/Ptau_396_ was significantly greater in those with the *TMEM106B* risk genotype compared to those without (*p* = 0.015; Table 3). However, there were no other significant associations after FDR correction. To determine whether *TMEM106B* risk alters the association between microglia and select cytokines previously found to be involved in neurodegenerative disease, we performed multiple linear regressions adjusting for age at death in those without and with the risk genotype. In those with the protective *TMEM106B* genotype, there were multiple significant associations between cytokines and markers of total microglia (IBA1), activated microglia (CD68), and TREM2-positive microglia (Fig. 3a). Cytokines IL-8 and IL-6 demonstrated significant positive associations with all three types of microglia. Additionally, IL-10 (p_IL-10_ = 0.004), IL-1β (p_IL-1β_ < 0.001), IL-4 (p_IL-4_ < 0.001), IL-8 (p_IL-8_ = 0.014), TNF-α (p_TNF-α_ = 0.009), TNF-β (p_TNF-β_ = 0.020), IL-1α (p_IL-1α_ = 0.041), and IL-6 (p_IL-6_ = 0.002) demonstrated positive associations with CD68. On the other hand, in the presence of the risk genotype, most of the cytokines’ associations with the microglial markers were lost (Fig. 3b). The only association that remained was between IL-6 and IBA1 density. Instead, IL-10 (p_IL-10_ = 0.009), IL-4 (p_IL-4_ = 0.007), and TNF-α (p_TNF-α_ = 0.006) demonstrated negative associations with TREM2. TNF-α (p_TNF-α_ = 0.004) was negatively associated with TREM2 in the risk genotype but trended toward a positive association with TREM2 in the protective genotype (p_TNF-α_ = 0.079). Sensitivity analyses in those aged 65 and younger showed similar results. Limiting the analyses to Caucasians only also showed similar effect sizes and maintained significant associations in IL-10, IL-1β, IL-4, IL-8, TNF-α, and IL-6 (Supplementary table c). Analyses within the AD-only cohort showed that cytokine associations with microglial markers were generally limited. Notably, the only overlapping association was observed for carriers of the risk allele: in AD, IL-6 was negatively associated with IBA1 (*β* = −0.690, *p* = 0.028), whereas in CTE, the association was positive (*β* = 0.527, *p* = 0.026). This contrast highlights differential cytokine–microglial marker relationships across disease contexts (Supplementary figure d).

**Table 3:** Neuropathology and neuroinflammatory cellular and cytokine measurements stratified by *TMEM106B* risk in CTE

	G:G; C:G alleles			C:C allele			P value
	N	Mean	SEM	N	Mean	SEM	
Neurodegenerative measures							
AT8 Cortex (AT8+ cell/mm2)	72	2.12	0.62	34	3.15	0.79	0.327
Total Tau (μg/g)	82	545.1	88.88	36	625.1	146.7	0.630
Phosphorylated Tau (μ/g)							
Ptau_181_	81	50440	2659	36	45050	4837	0.296
Ptau_202_	79	2257000	661000	34	3911000	1072000	0.181
Ptau_231_	82	1317000	304100	35	760300	499900	0.423
Ptau_396_	81	19640	3972	36	12320	6495	0.322
Ptau_202_/Ptau_396_	80	249.4	121.8	33	807.9	189.0	0.015
Ptau_231_/ Ptau_396_	80	217.7	58.72	36	353.8	82.99	0.192
Beta Amyloid(pg/mg)							
Aβ38	82	45.37	16.88	35	47.16	29.58	0.956
Aβ40	82	114.4	48. 14	35	95.58	69.52	0.828
Aβ42	81	322.7	51.70	36	312.4	76.55	0.912
Microglial markers							
IBA1 (IBA+ cell/mm^2^)	41	174.2	8.05	17	166.8	49.20	0.614
CD68 (CD68+ cell/mm^2^)	54	148.1)	8.05	20	156.5	15.88	0.611
TREM2 (ng/g)	60	40.85	176.6	24	303.9	158.8	0.151
Cytokines (pg/g)							
IFNγ	80	7.40	0.46	35	6.95	0.66	0.584
IL10	79	1.86	0.25	36	1.65	0.38	0.642
IL13	79	17.96	0.95	36	17.12	1.36	0.615
IL1β	79	26.22	5.07	36	8.37	9.30	0.071
IL4	79	1.23	0.10	36	1.21	0.14	0.880
IL8	79	180.2	41.30	36	84.77	60.84	0.198
TNFα	79	3.23	0.30	36	3.09	0.38	0.778
TNFβ	79	0.98	0.09	36	0.95	0.12	0.873
IL1α	80	7.84	0.47	35	7.67	0.78	0.850
IL6	79	56.11	19.37	36	3.23	30.38	0.137

**Fig. 4:**
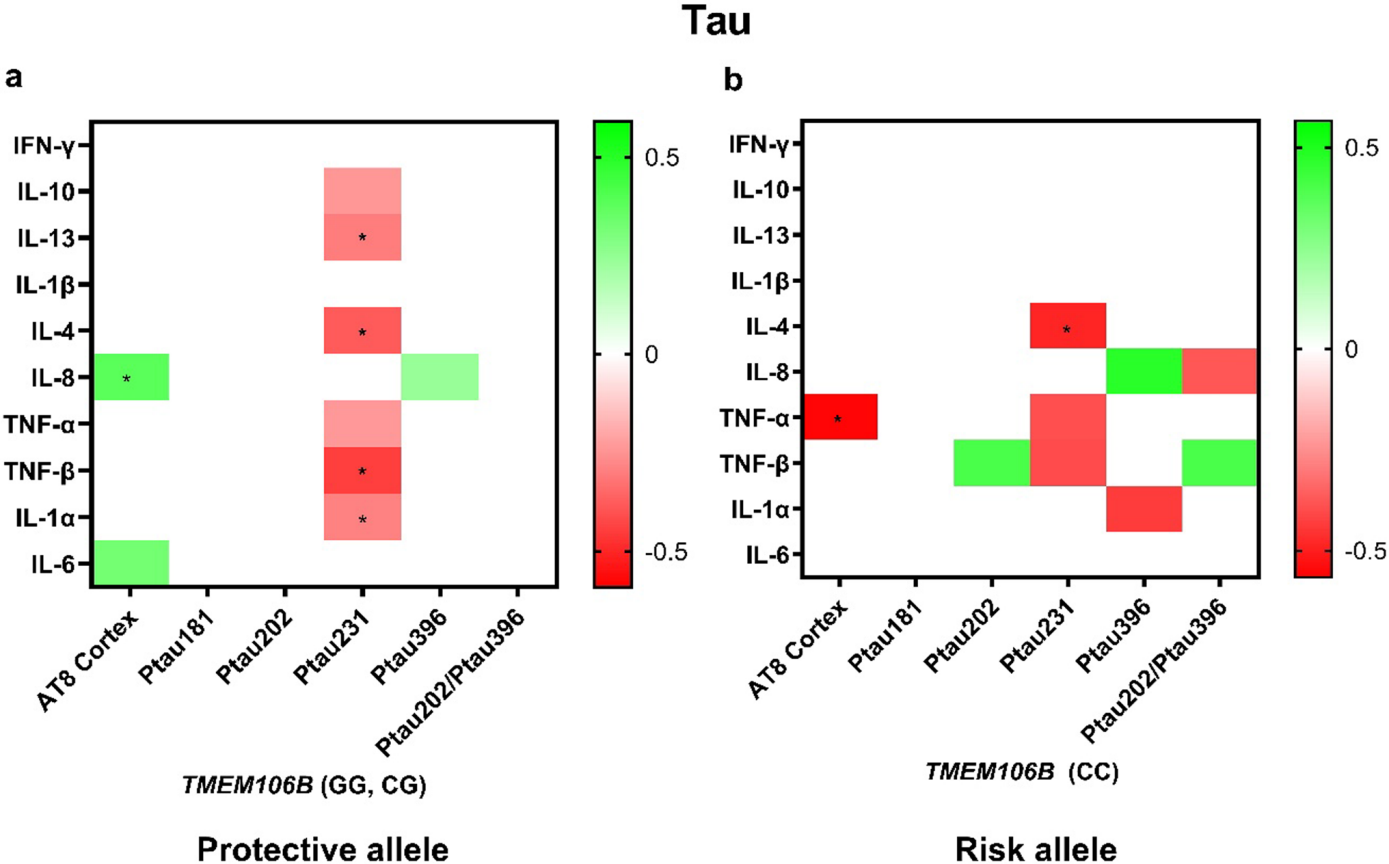
Cytokine associations with tau pathology vary by *TMEM106B* risk in CTE. Multiple linear regression analyses adjusted for age and APOE ε4 tested associations between inflammatory cytokines and measures of tau pathology including tau inclusions (AT8) levels of various phosphorylated tau species within the protective genotype (**a**) and the risk genotype (**b**). Heatmaps show *β* values with *p* < 0.05 (red-to-green shading indicated by key). *p < 0.05 after FDR adjustment

We next tested for associations between cytokines and measures of tau pathology in those without and with *TMEM106B* risk (Fig. 4). With the protective genotype, IL-10 (p_IL-10_ = 0.040), IL-13 (p_IL-13_ = 0.009), IL-4 (p_IL-4_ < 0.001), TNF-α (p_TNF-α_ = 0.039), TNF-β (p_TNF-β_ < 0.001), and IL-1α (p_IL-1α_ = 0.012) displayed negative associations with Ptau_231_. Additionally, IL-6 and IL-8 demonstrated positive associations with AT8 with the protective genotype. In contrast, in the presence of the risk genotype, TNF-β was positively associated with Ptau_202_:Ptau_396_ (*p* = 0.014), while IL-8 was negatively associated with Ptau_202_:Ptau_396_ (*p* = 0.034). TNF-α also demonstrated a negative association with AT8 in the risk genotype (*p* = 0.005). In the sensitivity analysis in the Caucasian subgroup, IL-13, IL-4, IL-8, TNF-β, and IL-6 maintained significant associations with their respective tau markers, while the other cytokines maintained similar effect sizes (Supplementary table e). In contrast to the associations observed in CTE, an analysis of an AD-only cohort revealed distinct patterns. Among carriers of the protective allele, IL-10, IL-4, TNF-α, and TNF-β were not associated with Ptau231 in AD, whereas these negative associations were present in CTE. In risk allele carriers, the negative association between IL-1α and Ptau396 are evident in CTE but absent in AD (Supplementary Figure f).

**Fig. 5:**
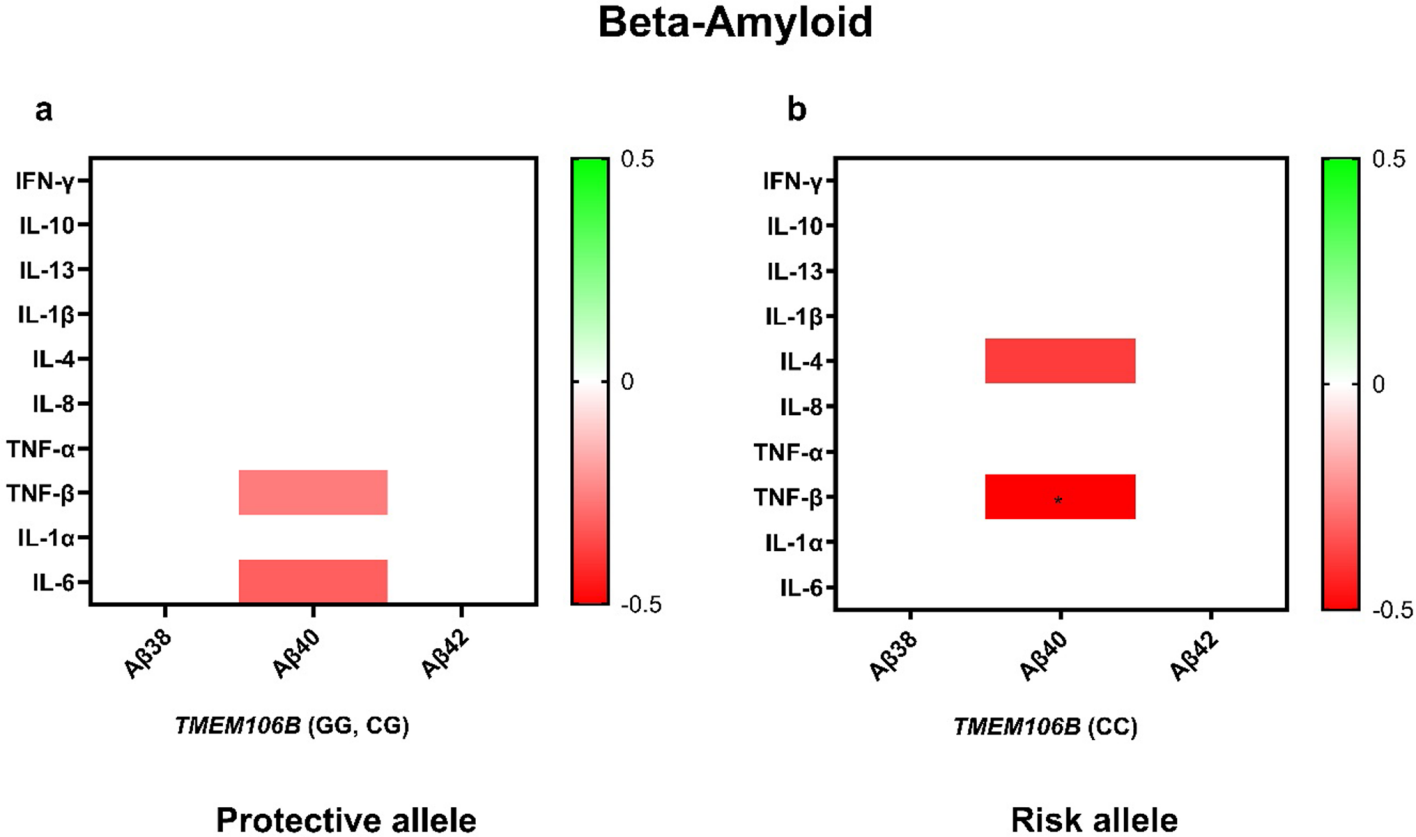
Effect of *TMEM106B* gene variation on the cytokine interaction beta-amyloid. Cytokine associations with beta-amyloid pathology vary by *TMEM106B* risk in CTE. Multiple linear regression analyses adjusted for age and APOE ε4 tested associations between inflammatory cytokines and beta-amyloid levels within the protective genotype (**a**) and the risk genotype (**b**). Heatmaps show *β* values with *p* < 0.05 (red-to-green shading indicated by key). *p < 0.05 after FDR adjustment

Accumulation of beta-amyloid plaques is a common comorbid pathology in CTE even in the absence of AD [51]. Therefore, we next tested for associations between cytokines and beta-amyloid levels in those without and with *TMEM106B* risk (Fig. 5). In those with the *TMEM106B* risk genotype, TNF-β (*p* = 0.003) and IL-4 (*p* = 0.027) were negatively associated with Aβ40 (Fig. 5). In the Caucasian subgroup sensitivity analysis, IL-10 shows a new association with Aβ40 in the risk allele (*β* = −0.349, *p* = 0.045), while the other risk allele associations remain significant with consistent effect sizes. In contrast, associations in the protective allele lose significance but retain similar effect sizes (Supplementary table g). In an AD-only cohort, cytokine associations with amyloid pathology were nearly exclusively positive, in contrast to those observed in CTE. Specifically, among carriers of the *TMEM106B* risk allele, IL-10 showed a positive association with Aβ40 (*β* = 0.449, *p* = 0.038) in AD, whereas the same genotype in the CTE cohort trended toward a negative association (*β* = −0.319, *p* = 0.068). Furthermore, for those with the protective allele, IFN-γ and IL-10 were positively associated with Aβ38 and Aβ40 in AD, while no significant associations with these amyloid markers were found in CTE (Supplementary Figure h).

## Discussion

In a group of donors with a history of RHI, the risk genotype of *rs3173615*, a coding single-nucleotide pleomorphism (SNP) in *TMEM106B*, was associated with more severe CTE and the presence of TDP-43 inclusions in those > 65 years. In those < = 65, *TMEM106B* risk was related to the presence of dementia. Within the dorsolateral prefrontal cortex, the *TMEM106B* risk variant modified the association between cytokines and microglia, as well as between cytokines and tau and beta-amyloid pathologies. This altered inflammatory association with pathology may partially mediate the pathological and clinical effects of *TMEM106B* variation in CTE.

The TMEM106B protein product is a glycoprotein predominantly localized in the membranes of endosomes and lysosomes [8]. SNP *rs1990622* on chromosome 7p21 has been repeatedly implicated as a genetic risk factor for FTLD-TDP and recently for AD [20, 46]. *Rs1990622* is in perfect linkage disequilibrium with only the SNP coding variant *rs3173615* (p.T185S) where the highly conserved threonine, coded by the common C genotype (Thr185: risk genotype) is replaced by a serine (Ser185: protective genotype) in the presence of the rarer G genotype. The serine replacement results in the faster degradation of the protein product [52]. Increased TMEM106B protein levels, resulting from the risk genotype, lead to lysosomal defects, including improper lysosomal formation, reduced lysosomal acidification and transport, and increased lysosomal stress [8, 46]. These lysosomal defects are hypothesized to result from impaired glycosylation at the crucial residues N183 and N256 [28]. The former is two residues away from the *rs3173615*. A recent genome-wide association study (GWAS) for gene variants found that *rs3173615* could have a functional role in the aging brain’s transcriptome [57]. Furthermore, the SNP is localized within a recently discovered 135 amino acid filament that originates from the C terminus of the TMEM106B protein which is common to neurodegenerative diseases characterized by tau, TDP-43, and α-synuclein [10]. Recently, the T185 haplotype has been associated with an increase of this filament in the frontal cortex and the accumulation of the filament has been linked to TDP-43 dysfunction [32]. Furthermore, *TMEM106B* T185 is associated with multiple TDP-43 pathologies (FTLD-TDP, ALS, and LATE-NC) [54, 55]. We recently reported a similar association with T185 and more severe tau pathology in CTE [12]. However, the mechanisms underlying this increased risk are largely unknown.

In individuals > 65 years of age, the *TMEM106B* risk was associated with an increased CTE stage and the presence of TDP-43 inclusions. The presence of the risk genotype more than doubled the odds of increasing one CTE stage such that the effect of having the *TMEM106B* risk genotype on CTE severity is equal to playing more than 8 years of contact sports. This is similar to the impact of APOE ε4 on CTE severity in American football players [4]. On the other hand, in those < = 65 years of age, *TMEM106B* risk was associated with increased odds of dementia and was equivalent to 27 years of playing contact sports. Surprisingly, TMEM106B risk was not associated with dementia in the older group, which may be due to the more potent effects of other age-related pathologies such as limbic TDP-43 inclusions or moderate-to-severe vascular disease, including CAA, arteriolosclerosis, or microinfarcts (Table 1). In addition, the association with dementia in the younger group appears to be independent of tau and TDP-43 pathology, suggesting that different mechanisms may contribute to cognitive impairment such as neuroinflammation.

*TMEM106B* gene variation is associated with TDP-43 inclusions in a various diseases, including FTLD-TDP and limbic age-related TDP-43 encephalopathy neuropathologic change (LATE-NC) [44]. TDP-43 inclusions also frequently occur in CTE and are associated with the duration of contact sports play, the stage of CTE, and age [47]. Here, we found that *TMEM106B* risk increased the odds of having TDP-43 inclusions in an older group with a history of RHI. Cyro-EM studies have found that TDP-43 inclusions in FTLD-TDP are primarily composed of TMEM106B protein filaments [21], a feature that may also be present in many TDP-43 proteinopathies [49]. Marks et al. recently showed that the accumulation of the TMEM106B filament is associated with TDP-43-dependent mis-splicing and pathology in FTLD-TDP [32]. Future studies should examine whether a similar mechanism of TDP-43 mis-splicing occurs in CTE [43].

**Fig. 6:**
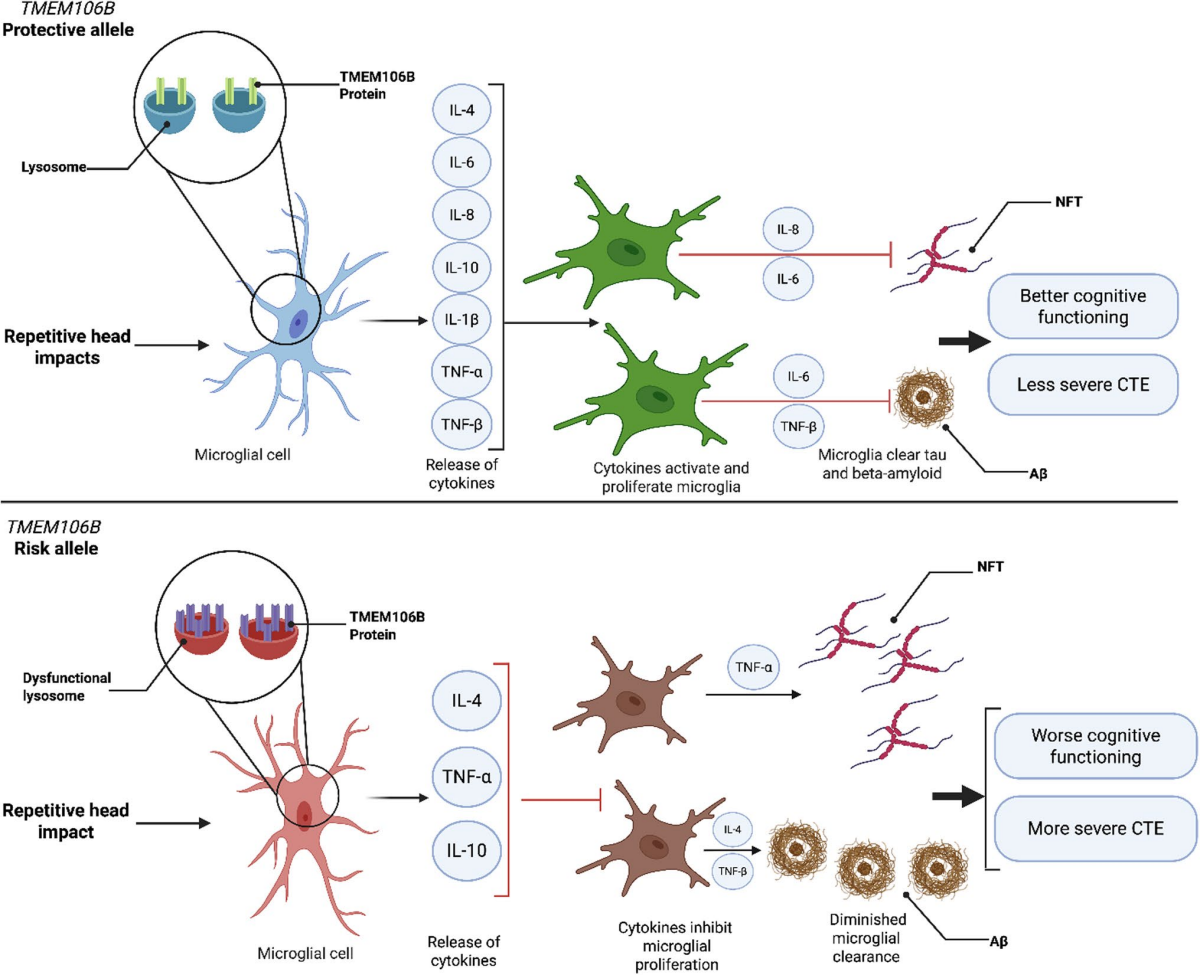
Schematic summary of the altered associations between cytokines, microglia, and pathology with *TMEM106B* risk in CTE. The neuroinflammatory associations (*p* < 0.05) are shown in those with the *TMEM106B* protective genotype (top panel) and in those with the risk genotype (bottom panel). The cytokines featured in the “Release of cytokines” step are those associated with IBA1, CD68, and TREM2. The cytokines associated with the neurofibrillary tangles (NFT) are those associated with tau pathology (AT8) in the cortex, while the cytokines associated with Aβ are cytokines that associated with both Aβ40 and Aβ42. The arrows show positive associations, and red bars illustrate negative associations. Created with BioRender.com

In fact, *TMEM106B* risk significantly altered the association of numerous inflammatory cytokines with microglia phenotypes and pathology. With the protective genotype, nearly all the tested cytokines had significant positive associations with microglia density. In particular, the most significant number of associations were with CD68 + cells, a marker of activated microglia and macrophages. In addition, IL-6 and IL-8 were significantly associated with all three microglia markers (CD68, IBA1, and TREM2). On the other hand, we found that in those with CTE and *TMEM106B* risk genotype, the associations between cytokines and microglia densities were lost and in the case of TREM2 significantly reversed. TREM2 may be a marker of disease-associated microglia (DAM) that may mitigate pathologies [25]. Previous studies have found increased gene expression of *TREM2* following traumatic brain injury [9, 29, 30], and in former American football players, higher levels of soluble Trem2 were associated with higher tau concentrations in the CSF [2]. Therefore, altered regulation of TREM2 + microglia, associated with *TMEM106B* risk, may promote worse pathology and clinical outcomes in CTE (Fig. 6).

Cytokine associations with tau pathology were also altered by *TMEM106B* risk. Specifically, tau tangle pathology as measured by AT8 immunohistochemistry was positively associated with IL-8 in those with the protective genotype, but this was lost with the risk genotype where instead TNF-α was negatively associated with AT8 tau pathology. These changes may underlie a decreased cytokine response to tau pathology in individuals with the *TMEM106B* risk genotype which may partially contribute to the greater accumulation of neurofibrillary tangles (Fig. 6). On the other hand, multiple cytokines demonstrated significant negative associations with Ptau_231_ in individuals carrying the protective genotype. In the risk genotype, the ratio of Ptau_202_ to Ptau_396_, which was previously shown to be increased in CTE compared to AD [50], was associated with increased TNF-β.

There were few significant cytokine associations with Aβ levels. IL-6 was negatively associated with Aβ-40 levels in individuals carrying the protective genotype, but not in those carrying the risk genotype. TNF-β was negatively associated with Aβ-40 with both protective and risk genotypes, and the association was most significant with the risk genotype. Beta-amyloid is associated with progression of CTE with diffuse plaques present in approximately 50% of cases and neuritic plaques present in ~ 30% [51]. Nevertheless, IL-6 may help mediate a DAM response to Aβ with the TMEM106B protective genotype that does not occur with the risk genotype in CTE (Fig. 6).

Although studies on repetitive head impacts are lacking, previous studies have found associations with IL-6 and IL-8 and traumatic brain injury. IL-6 has been positively associated with acute concussions in a veteran population [17]. Furthermore, a continued elevation of IL-6 in the CSF has been associated with worse Glasgow Outcome Scale scores after TBI [26]. Additionally, levels of IL-6 and IL-8 were found to increase after TBI in both plasma and CSF, and it has been suggested that IL-8 is a critical mediator of neuroinflammation [27, 31]. The prominent associations between IL-6 and IL-8 and pathology with the *TMEM106B* protective genotype are lost with the risk genotype, potentially allowing for greater pathological severity.

The study has several limitations. CTE donors were mostly self-selected or referred by the next-of-kin after death and are not representative of all individuals who have CTE. Previous data demonstrate that selection bias should only bias a genetic relationship if there are pleiotropic effects that influence selection in the study [40]. Methods for determining RHI exposure and clinical and medical history relied on retrospective review and inaccuracies associated with informant-report may introduce measurement error. By necessity sample sizes varied when comparing the effect of *TMEM106B* gene variation and as a result, effect estimates, and p values should be interpreted together and with caution. Having no female CTE donors prevented analyses to determine whether differences in sex contribute to cytokine associations with CTE-related outcomes. Sufficient genetic data were not available to account for population substructure, which could confound a genetic relationship. Our recent *TMEM106B* study was conducted in a Caucasian only population, and so, we ran a sensitivity analysis in the Caucasian subset of our donors and found similar associations and effect sizes. Future studies will be needed to understand better the effects of *rs3173615* in non-Caucasian ethnicities and in women.

## Conclusions

In a pathological group with a history of RHI the *TMEM106B* risk genotype was associated with worse CTE and TDP-43 inclusions in older individuals and increased risk of dementia in younger individuals, suggesting distinct mechanisms influenced by this variant across the lifespan. An aberrant neuroinflammatory response may partially mediate these effects as the *TMEM106B* risk genotype alters the neuroinflammatory association with both microglia and neurodegenerative pathologies in CTE. These findings underscore the importance of TMEM106B as a potential therapeutic target and warrant further research into its role in neuroinflammatory processes and disease progression in CTE and related diseases.

## Supplementary Information

Below is the link to the electronic supplementary material.

Supplementary file1 (DOCX 377 KB)

Supplementary file2 (DOCX 19 KB)

Supplementary file3 (DOCX 30 KB)

## Data Availability

Data used in this study are available from the Boston University Alzheimer’s Disease Center (https://www.bu.edu/alzresearch/information-for-investigators/) and from the authors by request.
