# Supplementary material for: Genetic variation in TMEM106B alters microglial activation and cytokine responses in chronic traumatic encephalopathy
Source: Acta Neuropathol. 2025 Nov 20;150(1):54. doi: 10.1007/s00401-025-02955-7 (PMC12634763; doi:10.1007/s00401-025-02955-7)
Supplement: Supplementary file 1 — Supplementary file1 (DOCX 377 KB) [file 401_2025_2955_MOESM1_ESM.docx]

| **Supplementary Table a:** Demographic, Clinical, Genetic, and Neuropathological Characteristics for AD cohort | | |
| --- | --- | --- |
| **Characteristics** | | AD (n=51) |
| Sex (Male/Female) | | 26/25 |
| Age median (IQR), | | 90 (10) |
| RHI years median (IQR) | | 9.5 (7.25) |
| Age of First Contact Sports Exposure median (IQR) | | 11.0 (6.0) |
| Sport | Football | 11 (26.2%) |
|  | Hockey | 2 (4.9%) |
|  | Other | 2 (4.9%) |
| *TMEM106B* (CC**/**G+)- | | 16 (31.4%) |
| *APOE E4 status % (Y/N)* | | 27 (47.1%) |
| TDP-43 % (Y/N) | | 5 (45.5%) |
| Dementia status % (Y/N) | | 6 (25.0%) |
| Brainstem Lewy Body pathology %Y (Y/N) | | 6 (54.5%) |
| **Alzheimer disease neuropathologic change** | | |
| Braak Stage | 0 | 0 (0%) |
|  | I-II | 0 (0%) |
|  | III-IV | 17 (36.2%) |
|  | V-VI | 30 (63.8%) |
| CERAD Score | C0 | 0 (0%) |
|  | C1 | 22 (46.8%) |
|  | C2 | 22 (46.8%) |
|  | C3 | 3 (6.4%) |
| Remote microinfarcts % (Y/N) | | 22 (53.2%) |
| Cerebral Amyloid Angiopathy (moderate-severe) | | 19 (40.4%) |

Supplementary Table a: Not all cases had available data for each variable; denominators vary by measure.)

**b.**

COMPUTE LL_full = -285.722 / 2. /* Final model */

COMPUTE LL_null = -310.068 / 2. /* Intercept-only model */

COMPUTE N = 110. /* Sample size */

* --- Compute Cox & Snell R² ---.

COMPUTE R2_CoxSnell = 1 - EXP((-2 * (LL_full - LL_null)) / N).

* --- Compute Nagelkerke R² ---.

COMPUTE R2_Nagelkerke = R2_CoxSnell / (1 - EXP((2 * LL_null) / N)).

* --- Compute Cohen's f² ---.

COMPUTE f2 = R2_Nagelkerke / (1 - R2_Nagelkerke).

* --- Format numbers for readability ---.

FORMATS R2_CoxSnell R2_Nagelkerke f2 (F8.3).

* --- Display the results in the output window ---.

DISPLAY R2_CoxSnell R2_Nagelkerke f2.

EXECUTE.

**Supplementary figure b:** SPSS code demonstrating the calculation for effect size that was used for Power analysis in G*Power (version 3.1.9.7).

**c.**

| **CTE** |  |  |  |  |  |  |
| --- | --- | --- | --- | --- | --- | --- |
| **IFNg** | **N** | **CD68C** | **N** | **IBA1C** | **N** | **TREM2** |
| **GG, CG** | 38 | .283 (.169) | 31 | .345 | 46 | .218 |
| **CC** | 19 | .122 | 16 | .540 | 23 | **.049 (-.423)** ▲ |
| **IL10** |  |  |  |  |  |  |
| **GG, CG** | 37 | **.013 (.387)** ● | 31 | .914 | 45 | .553 |
| **CC** | 20 | .441 | 17 | .250 | 24 | **.003 (-.562)** ● |
| **IL13** |  |  |  |  |  |  |
| **GG, CG** | 37 | .871 | 30 | **.042 (-.377)** ▲ | 45 | .805 |
| **CC** | 20 | .200 | 17 | .057 (-.486) | 24 | .911 |
| **IL1B** |  |  |  |  |  |  |
| **GG, CG** | 37 | **.003 (.476)** ● | 30 | **.038 (.387)** ● | 45 | .803 |
| **CC** | 20 | .318 | 17 | .626 | 24 | .087 (-.366) |
| **IL4** |  |  |  |  |  |  |
| **GG, CG** | 37 | **.008 (.412)** ● | 31 | .469 | 45 | .065 (.266) |
| **CC** | 20 | .322 | 17 | **.049 (-.501)** ▲ | 24 | **.003 (-.593)** ● |
| **IL8** |  |  |  |  |  |  |
| **GG, CG** | 38 | **.031 (.350)** ● | 31 | **.018 (.411)** ● | 46 | **.003 (.416)** ● |
| **CC** | 20 | .976 | 17 | .052 (.495) | 24 | .898 |
| **TNF-α** |  |  |  |  |  |  |
| **GG, CG** | 37 | **.025 (.357)** ● | 30 | .179 | 45 | .052 (.279) |
| **CC** | 20 | .955 | 17 | .436 | 24 | **.004 (-.575)** ● |
| **TNF-Β** |  |  |  |  |  |  |
| **GG, CG** | 38 | .080 (.281) ■ | 31 | .461 | 46 | .931 |
| **CC** | 20 | .163 | 17 | .846 | 24 | **.018 (-.483)** ▲ |
| **IL1A** |  |  |  |  |  |  |
| **GG, CG** | 38 | .327 (.166) ■ | 31 | .276 | 46 | .989 |
| **CC** | 19 | .126 | 17 | .735 | 23 | .551 |
| **IL6** |  |  |  |  |  |  |
| **GG, CG** | 38 | **.003 (.468)** ● | 31 | .075 (.325) ■ | 46 | **.007 (.394)** ● |
| **CC** | 20 | .117 | 17 | **.021 (.528)** ● | 24 | .848 |

**Supplementary table c:** Effect of *TMEM106B* gene variation on cytokine association with microglial levels in CTE in a Caucasian-only subgroup. Cytokine associations with microglia subtypes vary by *TMEM106B* risk in CTE. Multiple linear regression analyses adjusted for age tested associations between inflammatory cytokines and microglial markers within the protective genotype and the risk genotype. Values are presented as p value (β value). Legend: ■ Lost significance; ● Maintained significance; ▲ Became newly significant.

**d.**


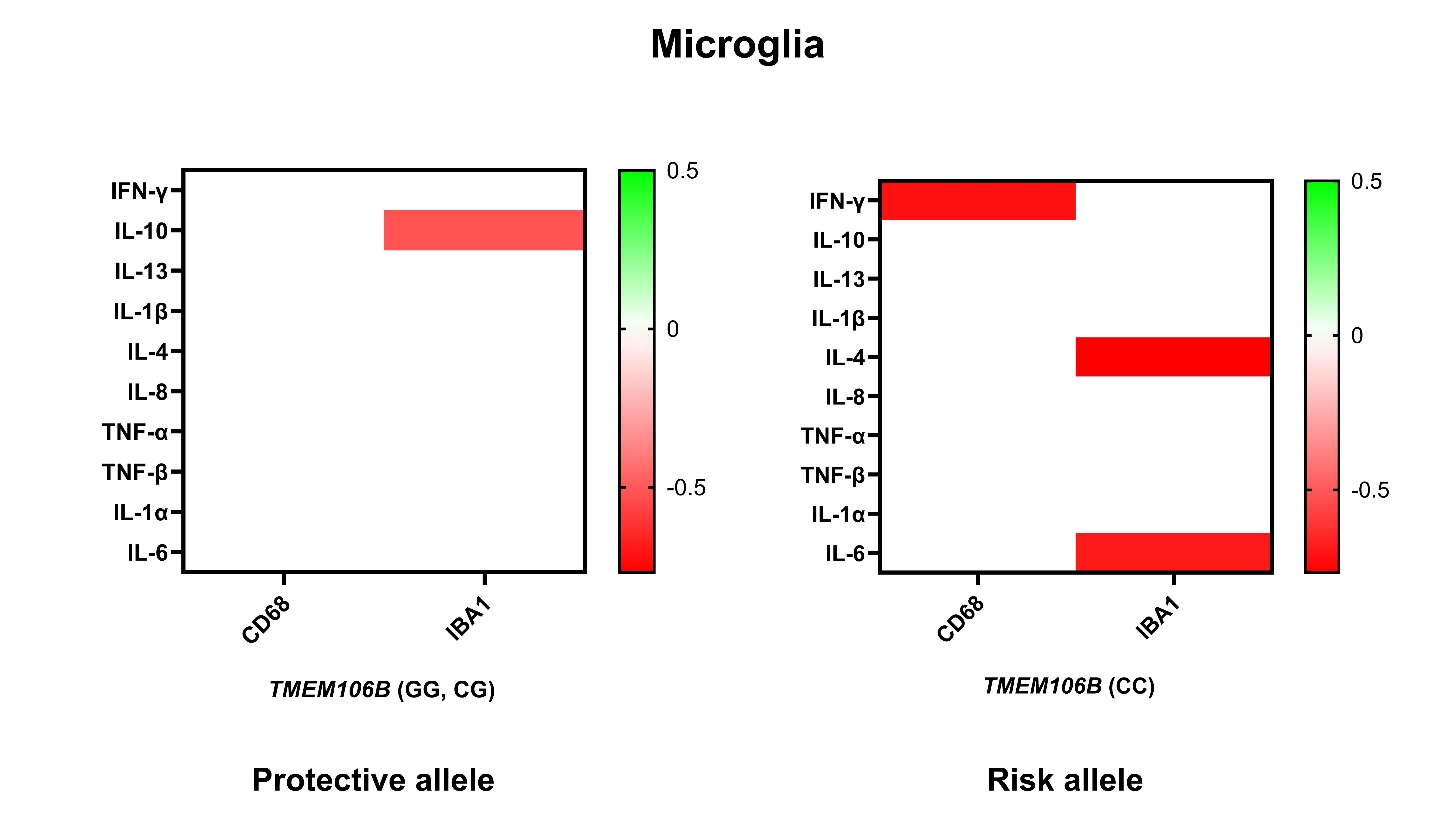


**Supplementary figure d:** Effect of *TMEM106B* gene variation on microglial levels in AD. Cytokine associations with microglia subtypes vary by *TMEM106B* risk in CTE. Multiple linear regression analyses adjusted for age, gender and APOE ε4 tested associations between inflammatory cytokines and microglial markers within the protective genotype (a) and the risk genotype (b). Heatmaps show β values with p<0.05 (red to green shading indicated by key). *p<0.05 after FDR adjustment.

**e.**

| **CTE** |  |  |  |  |  |  |  |  |  |  |  |  |
| --- | --- | --- | --- | --- | --- | --- | --- | --- | --- | --- | --- | --- |
| **IFNg** | **N** | **AT8 Cort** | **N** | **Ptau181** | **N** | **Ptau202** | **N** | **Ptau231** | **N** | **Ptau396** | **N** | **Ptau202/Ptau396** |
| **GG, CG** | 56 | .097 | 60 | .980 | 57 | .099 | 60 | .550 | 59 | .266 | 58 | .726 |
| **CC** | 33 | .407 | 35 | .706 | 33 | .193 | 34 | .314 | 35 | .517 | 33 | .063 |
| **IL10** |  |  |  |  |  |  |  |  |  |  |  |  |
| **GG, CG** | 56 | .917 | 59 | .772 | 56 | .869 | 59 | .066 (-.241)■ | 58 | .973 | 57 | .426 |
| **CC** | 34 | .180 | 36 | .980 | 34 | .306 | 35 | .101 | 36 | .116 | 33 | .569 |
| **IL13** |  |  |  |  |  |  |  |  |  |  |  |  |
| **GG, CG** | 55 | .110 | 59 | .091 | 56 | .460 | 59 | **.003 (-.378)**● | 58 | .778 | 57 | .629 |
| **CC** | 34 | .786 | 36 | .097 | 34 | .259 | 35 | .053 | 36 | .208 | 33 | .080 |
| **IL1B** |  |  |  |  |  |  |  |  |  |  |  |  |
| **GG, CG** | 55 | .494 | 59 | .741 | 56 | .874 | 59 | .555 | 58 | .776 | 57 | .554 |
| **CC** | 34 | .906 | 36 | .659 | 34 | .232 | 35 | .494 | 35 | .851 | 33 | .419 |
| **IL4** |  |  |  |  |  |  |  |  |  |  |  |  |
| **GG, CG** | 56 | .935 | 59 | .297 | 56 | .161 | 59 | **.006 (-.348)**● | 58 | .537 | 57 | .472 |
| **CC** | 34 | .083 | 36 | .585 | 34 | .179 | 35 | **.004 (-.476)**● | 36 | **.036 (-.433)**▲ | 33 | .062 |
| **IL8** |  |  |  |  |  |  |  |  |  |  |  |  |
| **GG, CG** | 56 | **.005 (.417)**● | 60 | .449 | 57 | .273 | 60 | .925 | 59 | .112 (.207)■ | 58 | .473 |
| **CC** | 34 | .158 | 36 | .255 | 34 | .856 | 35 | .567 | 36 | .066 | 33 | .069 (-.320)■ |
| **TNF-α** |  |  |  |  |  |  |  |  |  |  |  |  |
| **GG, CG** | 55 | .933 | 59 | .524 | 56 | .970 | 59 | .178 (-.174) ■ | 58 | .737 | 57 | .647 |
| **CC** | 34 | **.003 (-.580)**● | 36 | .770 | 34 | .962 | 35 | **.019 (-.403)**● | 36 | **.036 (-.437)**▲ | 33 | .593 |
| **TNF-Β** |  |  |  |  |  |  |  |  |  |  |  |  |
| **GG, CG** | 56 | .266 | 60 | .236 | 57 | .759 | 60 | **<.001 (-.448)**● | 59 | .632 | 58 | .312 |
| **CC** | 34 | .369 | 36 | .594 | 34 | **.035 (.402)**● | 35 | **.014 (-.423)**● | 36 | .106 | 33 | **.014 (.430)**● |
| **IL1A** |  |  |  |  |  |  |  |  |  |  |  |  |
| **GG, CG** | 56 | .143 | 60 | .707 | 57 | .415 | 60 | **.089 (-.224)**■ | 59 | .307 | 58 | .360 |
| **CC** | 33 | .466 | 35 | .251 | 33 | .893 | 34 | .090 (-.297)■ | 35 | **.038 (-.429)**● | 32 | .639 |
| **IL6** |  |  |  |  |  |  |  |  |  |  |  |  |
| **GG, CG** | 56 | **.020 (.351)**● | 60 | .168 | 57 | .109 | 60 | .338 | 59 | .231 | 58 | .345 |
| **CC** | 34 | .173 | 36 | .316 | 34 | .415 | 35 | .169 | 36 | .091 | 33 | .300 |

**Supplementary table e:** Effect of *TMEM106B* gene variation on cytokine interaction on tau pathology in CTE in a Caucasian-only subgroup. Cytokine associations with tau vary by *TMEM106B* risk in CTE. Multiple linear regression analyses adjusted for age tested associations between inflammatory cytokines and microglial markers within the protective genotype and the risk genotype. Values are presented as p value (β value). Legend: ■ Lost significance; ● Maintained significance; ▲ Became newly significant.

**f.**


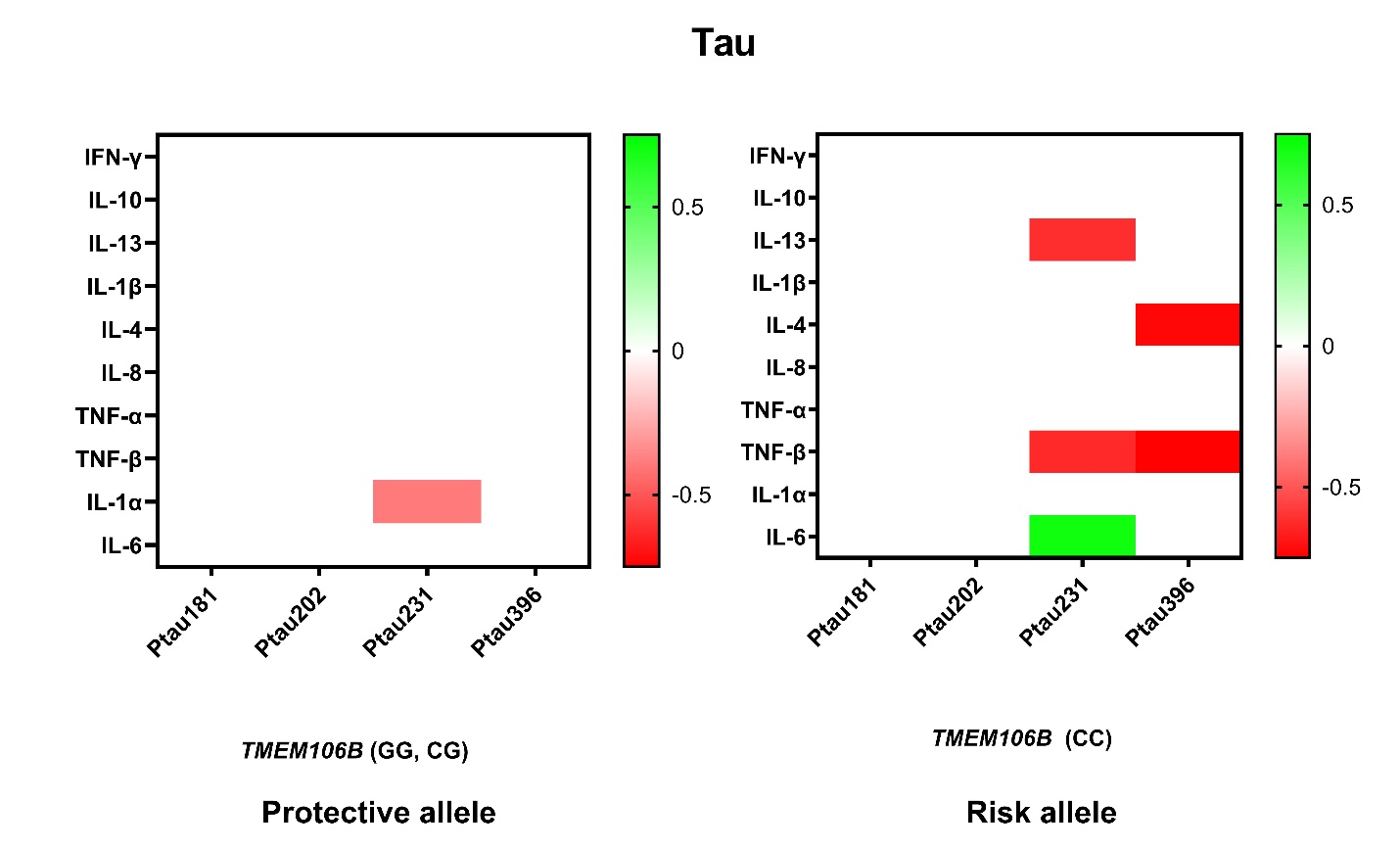


**Supplementary figure f:** Cytokine associations with tau pathology vary by *TMEM106B* risk in AD. Multiple linear regression analyses adjusted for age, gender and APOE ε4 tested associations between inflammatory cytokines and measures of tau pathology including tau inclusions (AT8) levels of various phosphorylated tau species within the protective genotype (a) and the risk genotype (b). Heatmaps show β values with p<0.05 (red to green shading indicated by key). *p<0.05 after FDR adjustment.

**g.**

| **CTE** |  |  |  |  |  |  |
| --- | --- | --- | --- | --- | --- | --- |
| **IFNg** | **N** | **AB38** | **N** | **AB40** | **N** | **AB42** |
| **GG, CG** | 60 | .337 | 60 | .390 | 59 | .146 |
| **CC** | 34 | .564 | 34 | .220 | 35 | .949 |
| **IL10** |  |  |  |  |  |  |
| **GG, CG** | 59 | .865 | 59 | .623 | 58 | .532 |
| **CC** | 35 | .320 | 35 | **.045 (-.349)**▲ | 36 | .706 |
| **IL13** |  |  |  |  |  |  |
| **GG, CG** | 59 | .630 | 59 | .346 | 58 | .897 |
| **CC** | 35 | .586 | 35 | .325 | 36 | .838 |
| **IL1B** |  |  |  |  |  |  |
| **GG, CG** | 59 | .460 | 59 | .942 | 58 | .966 |
| **CC** | 35 | .933 | 35 | .358 | 36 | .564 |
| **IL4** |  |  |  |  |  |  |
| **GG, CG** | 59 | .411 | 59 | .805 | 58 | .235 |
| **CC** | 35 | .572 | 35 | **.018 (-.402)**● | 36 | .423 |
| **IL8** |  |  |  |  |  |  |
| **GG, CG** | 60 | .554 | 60 | .228 | 59 | .443 |
| **CC** | 35 | .154 | 35 | .488 | 36 | .792 |
| **TNF-α** |  |  |  |  |  |  |
| **GG, CG** | 59 | .833 | 59 | .534 | 58 | .989 |
| CC | 35 | .622 | 35 | .075 | 36 | .285 |
| **TNF-Β** |  |  |  |  |  |  |
| **GG, CG** | 60 | .818 | 60 | .116 (-.208)■ | 59 | .379 |
| **CC** | 35 | .176 | 35 | **.001 (-.529)**● | 36 | .229 |
| **IL1A** |  |  |  |  |  |  |
| **GG, CG** | 60 | .295 | 60 | .135 (-.203)■ | 59 | .714 |
| **CC** | 34 | .189 | 34 | .096 | 35 | .955 |
| **IL6** |  |  |  |  |  |  |
| **GG, CG** | 60 | .572 | 60 | .110 (.217)■ | 59 | .147 (.203)■ |
| **CC** | 35 | .709 | 35 | .723 | 36 | .434 |

**Supplementary table g:** Effect of *TMEM106B* gene variation on Cytokine interaction on beta amyloid levels in CTE in a Caucasian-only subgroup. Cytokine associations with beta amyloid vary by *TMEM106B* risk in CTE. Multiple linear regression analyses adjusted for age tested associations between inflammatory cytokines and microglial markers within the protective genotype and the risk genotype. Values are presented as p value (β value). Legend: ■ Lost significance; ● Maintained significance; ▲ Became newly significant.

**h.**


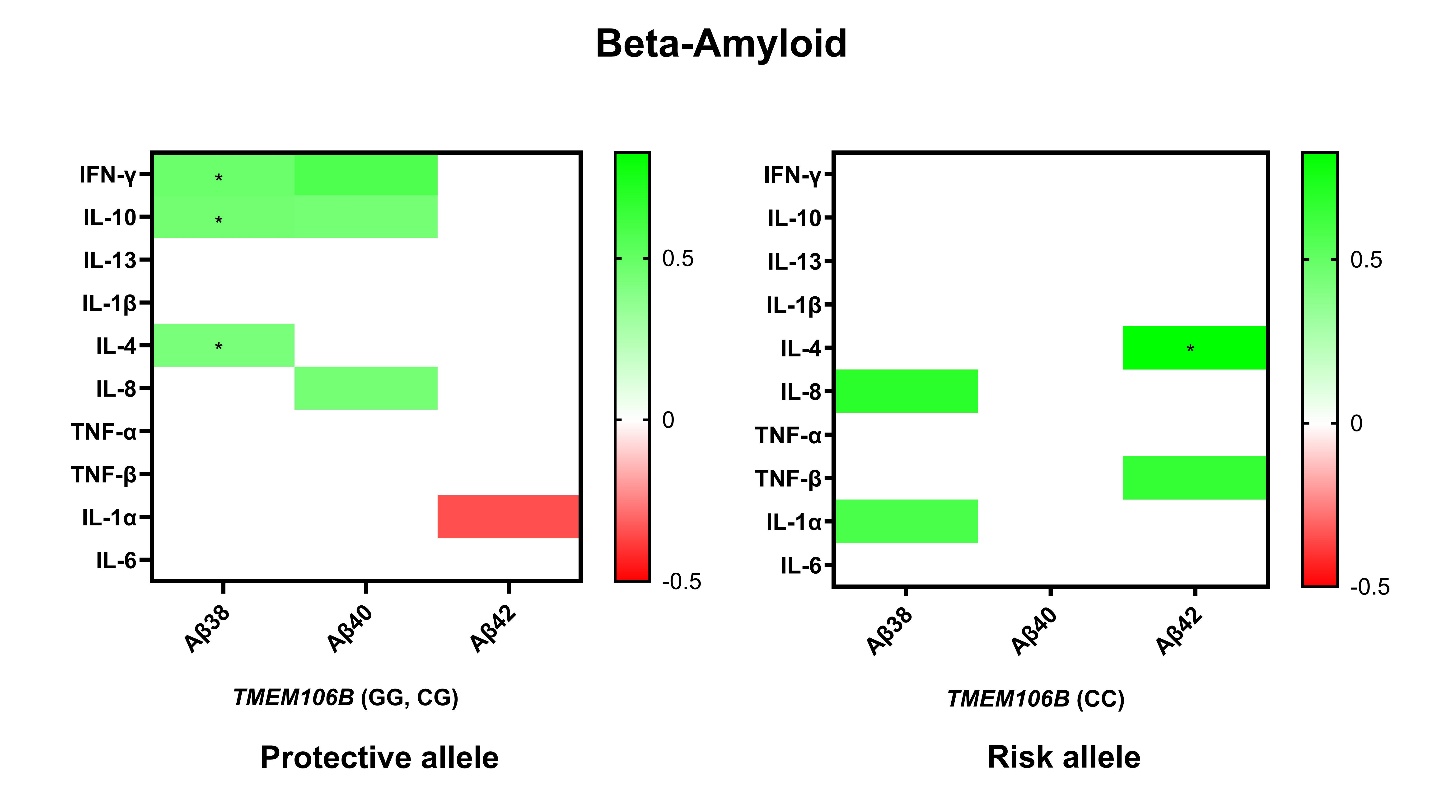


**Supplementary figure h:** Effect of *TMEM106B* gene variation on the cytokine interaction beta-amyloid. Cytokine associations with beta-amyloid pathology vary by *TMEM106B* risk in CTE. Multiple linear regression analyses adjusted for age and APOE ε4 tested associations between inflammatory cytokines and beta-amyloid levels within the protective genotype (a) and the risk genotype (b). Heatmaps show β values with p<0.05 (red to green shading indicated by key). *p<0.05 after FDR adjustment.
