# Supplementary material for: Genetic variation in TMEM106B alters microglial activation and cytokine responses in chronic traumatic encephalopathy
Source: Acta Neuropathol. 2025 Nov 20;150(1):54. doi: 10.1007/s00401-025-02955-7 (PMC12634763; doi:10.1007/s00401-025-02955-7)
Supplement: Supplementary file 2 — Supplementary file2 (DOCX 19 KB) [file 401_2025_2955_MOESM2_ESM.docx]

White population only; Box= Lost significance; Box= Maintained significance; Box= Became newly significant

| **CTE** |  |  |  |  |  |  |
| --- | --- | --- | --- | --- | --- | --- |
| **IFNg** | **N** | **CD68C** | **N** | **IBA1C** | **N** | **TREM2** |
| **TMEM=0** | 38 | .283 (.169) | 31 | .345 | 46 | .218 |
| **TMEM=1** | 19 | .122 | 16 | .540 | 23 | **.049 (-.423)** |
| **IL10** |  |  |  |  |  |  |
| **TMEM=0** | 37 | **.013 (.387)** | 31 | .914 | 45 | .553 |
| **TMEM=1** | 20 | .441 | 17 | .250 | 24 | **.003 (-.562)** |
| **IL13** |  |  |  |  |  |  |
| **TMEM=0** | 37 | .871 | 30 | **.042 (-.377)** | 45 | .805 |
| **TMEM=1** | 20 | .200 | 17 | .057 (-.486) | 24 | .911 |
| **IL1B** |  |  |  |  |  |  |
| **TMEM=0** | 37 | **.003 (.476)** | 30 | **.038 (.387)** | 45 | .803 |
| **TMEM=1** | 20 | .318 | 17 | .626 | 24 | .087 (-.366) |
| **IL4** |  |  |  |  |  |  |
| **TMEM=0** | 37 | **.008 (.412)** | 31 | .469 | 45 | .065 (.266) |
| **TMEM=1** | 20 | .322 | 17 | **.049 (-.501)** | 24 | **.003 (-.593)** |
| **IL8** |  |  |  |  |  |  |
| **TMEM=0** | 38 | **.031 (.350)** | 31 | **.018 (.411)** | 46 | **.003 (.416)** |
| **TMEM=1** | 20 | .976 | 17 | .052 (.495) | 24 | .898 |
| **TNFa** |  |  |  |  |  |  |
| **TMEM=0** | 37 | **.025 (.357)** | 30 | .179 | 45 | .052 (.279) |
| **TMEM=1** | 20 | .955 | 17 | .436 | 24 | **.004 (-.575)** |
| **TNFB** |  |  |  |  |  |  |
| **TMEM=0** | 38 | .080 (.281) | 31 | .461 | 46 | .931 |
| **TMEM=1** | 20 | .163 | 17 | .846 | 24 | **.018 (-.483)** |
| **IL1A** |  |  |  |  |  |  |
| **TMEM=0** | 38 | .327 (.166) | 31 | .276 | 46 | .989 |
| **TMEM=1** | 19 | .126 | 17 | .735 | 23 | .551 |
| **IL6** |  |  |  |  |  |  |
| **TMEM=0** | 38 | **.003 (.468)** | 31 | .075 (.325) | 46 | **.007 (.394)** |
| **TMEM=1** | 20 | .117 | 17 | **.021 (.528)** | 24 | .848 |
