# Supplementary material for: Genetic variation in TMEM106B alters microglial activation and cytokine responses in chronic traumatic encephalopathy
Source: Acta Neuropathol. 2025 Nov 20;150(1):54. doi: 10.1007/s00401-025-02955-7 (PMC12634763; doi:10.1007/s00401-025-02955-7)
Supplement: Supplementary file 3 — Supplementary file3 (DOCX 30 KB) [file 401_2025_2955_MOESM3_ESM.docx]

| **CTE** |  |  |  |  |  |  |
| --- | --- | --- | --- | --- | --- | --- |
| **IFNg** | **N** | **AB38** | **N** | **AB40** | **N** | **AB42** |
| **TMEM=0** | 60 | .337 | 60 | .390 | 59 | .146 |
| **TMEM=1** | 34 | .564 | 34 | .220 | 35 | .949 |
| **IL10** |  |  |  |  |  |  |
| **TMEM=0** | 59 | .865 | 59 | .623 | 58 | .532 |
| **TMEM=1** | 35 | .320 | 35 | **.045 (-.349)** | 36 | .706 |
| **IL13** |  |  |  |  |  |  |
| **TMEM=0** | 59 | .630 | 59 | .346 | 58 | .897 |
| **TMEM=1** | 35 | .586 | 35 | .325 | 36 | .838 |
| **IL1B** |  |  |  |  |  |  |
| **TMEM=0** | 59 | .460 | 59 | .942 | 58 | .966 |
| **TMEM=1** | 35 | .933 | 35 | .358 | 36 | .564 |
| **IL4** |  |  |  |  |  |  |
| **TMEM=0** | 59 | .411 | 59 | .805 | 58 | .235 |
| **TMEM=1** | 35 | .572 | 35 | **.018 (-.402)** | 36 | .423 |
| **IL8** |  |  |  |  |  |  |
| **TMEM=0** | 60 | .554 | 60 | .228 | 59 | .443 |
| **TMEM=1** | 35 | .154 | 35 | .488 | 36 | .792 |
| **TNFa** |  |  |  |  |  |  |
| **TMEM=0** | 59 | .833 | 59 | .534 | 58 | .989 |
| TMEM=1 | 35 | .622 | 35 | .075 | 36 | .285 |
| **TNFB** |  |  |  |  |  |  |
| **TMEM=0** | 60 | .818 | 60 | .116 (-.208) | 59 | .379 |
| **TMEM=1** | 35 | .176 | 35 | **.001 (-.529)** | 36 | .229 |
| **IL1A** |  |  |  |  |  |  |
| **TMEM=0** | 60 | .295 | 60 | .135 (-.203) | 59 | .714 |
| **TMEM=1** | 34 | .189 | 34 | .096 | 35 | .955 |
| **IL6** |  |  |  |  |  |  |
| **TMEM=0** | 60 | .572 | 60 | .110 (.217) | 59 | .147 (.203) |
| **TMEM=1** | 35 | .709 | 35 | .723 | 36 | .434 |

White population only adjusted for agedeath ; Box= Lost significance; Box= Maintained significance; Box= Became newly significant

| **CTE** |  |  |  |  |  |  |  |  |  |  |  |  |  |  |  |  |
| --- | --- | --- | --- | --- | --- | --- | --- | --- | --- | --- | --- | --- | --- | --- | --- | --- |
| **IFNg** | **N** | **AT8 Cort** | **N** | **AT8C** | **N** | **Ptau181** | **N** | **Ptau202** | **N** | **Ptau231** | **N** | **Ptau396** | **N** | **Ptau202/Ptau396** | **N** | **Ptau231/Ptau396** |
| **TMEM=0** | 56 | .097 | 52 | .478 | 60 | .980 | 57 | .099 | 60 | .550 | 59 | .266 | 58 | .726 | 58 | .134 (-.194) |
| **TMEM=1** | 33 | .407 | 30 | .760 | 35 | .706 | 33 | .193 | 34 | .314 | 35 | .517 | 33 | .063 | 35 | .351 |
| **IL10** |  |  |  |  |  |  |  |  |  |  |  |  |  |  |  |  |
| **TMEM=0** | 56 | .917 | 51 | .604 | 59 | .772 | 56 | .869 | 59 | .066 | 58 | .973 | 57 | .426 | 57 | .321 |
| **TMEM=1** | 34 | .180 | 31 | .560 | 36 | .980 | 34 | .306 | 35 | .101 | 36 | .116 | 33 | .569 | 36 | .399 |
| **IL13** |  |  |  |  |  |  |  |  |  |  |  |  |  |  |  |  |
| **TMEM=0** | 55 | .110 | 51 | .304 | 59 | .091 | 56 | .460 | 59 | **.003 (-.378)** | 58 | .778 | 57 | .629 | 57 | .770 |
| **TMEM=1** | 34 | .786 | 31 | .724 | 36 | .097 | 34 | .259 | 35 | .053 | 36 | .208 | 33 | .080 | 36 | .186 |
| **IL1B** |  |  |  |  |  |  |  |  |  |  |  |  |  |  |  |  |
| **TMEM=0** | 55 | .494 | 51 | .573 | 59 | .741 | 56 | .874 | 59 | .555 | 58 | .776 | 57 | .554 | 57 | .238 |
| **TMEM=1** | 34 | .906 | 31 | .897 | 36 | .659 | 34 | .232 | 35 | .494 | 35 | .851 | 33 | .419 | 36 | .651 |
| **IL4** |  |  |  |  |  |  |  |  |  |  |  |  |  |  |  |  |
| **TMEM=0** | 56 | .935 | 51 | .971 | 59 | .297 | 56 | .161 | 59 | **.006 (-.348)** | 58 | .537 | 57 | .472 | 57 | **.050 (-.254)** |
| **TMEM=1** | 34 | .083 | 31 | .425 | 36 | .585 | 34 | .179 | 35 | **.004 (-.476)** | 36 | **.036 (-.433)** | 33 | .062 | 36 | .228 |
| **IL8** |  |  |  |  |  |  |  |  |  |  |  |  |  |  |  |  |
| **TMEM=0** | 56 | **.005 (.417)** | 52 | .203 | 60 | .449 | 57 | .273 | 60 | .925 | 59 | .112 (.207) | 58 | .473 | 58 | **.**102 (-.215) |
| **TMEM=1** | 34 | .158 | 31 | .293 | 36 | .255 | 34 | .856 | 35 | .567 | 36 | .066 | 33 | .069 | 33 | .069 |
| **TNFa** |  |  |  |  |  |  |  |  |  |  |  |  |  |  |  |  |
| **TMEM=0** | 55 | .933 | 51 | .208 | 59 | .524 | 56 | .970 | 59 | .178 | 58 | .737 | 57 | .647 | 57 | .788 |
| **TMEM=1** | 34 | **.003 (-.580)** | 31 | **.026 (-.593)** | 36 | .770 | 34 | .962 | 35 | **.019 (-.403)** | 36 | **.036 (-.437)** | 33 | .593 | 36 | .565 |
| **TNFB** |  |  |  |  |  |  |  |  |  |  |  |  |  |  |  |  |
| **TMEM=0** | 56 | .266 | 52 | .200 | 60 | .236 | 57 | .759 | 60 | **<.001 (-.448)** | 59 | .632 | 58 | .312 | 58 | .129 |
| **TMEM=1** | 34 | .369 | 31 | .881 | 36 | .594 | 34 | **.035 (.402)** | 35 | **.014 (-.423)** | 36 | .106 | 33 | **.014 (.430)** | 36 | .284 |
| **IL1A** |  |  |  |  |  |  |  |  |  |  |  |  |  |  |  |  |
| **TMEM=0** | 56 | .143 | 52 | .178 | 60 | .707 | 57 | .415 | 60 | **.089 (-.224)** | 59 | .307 | 58 | .360 | 58 | .756 |
| **TMEM=1** | 33 | .466 | 30 | .612 | 35 | .251 | 33 | .893 | 34 | .090 | 35 | **.038 (-.429)** | 32 | .639 | 35 | .887 |
| **IL6** |  |  |  |  |  |  |  |  |  |  |  |  |  |  |  |  |
| **TMEM=0** | 56 | **.020 (.351)** | 52 | .158 (.239) | 60 | .168 | 57 | .109 | 60 | .338 | 59 | .231 | 58 | .345 | 58 | **.065 (-.246)** |
| **TMEM=1** | 34 | .173 | 31 | .598 | 36 | .316 | 34 | .415 | 35 | .169 | 36 | .091 | 33 | .300 | 36 | .801 |
